# Integrative multi-omics networks identify PKCδ and DNA-PK as master kinases of glioblastoma subtypes and guide targeted cancer therapy

**DOI:** 10.1038/s43018-022-00510-x

**Published:** 2023-02-02

**Authors:** Simona Migliozzi, Young Taek Oh, Mohammad Hasanain, Luciano Garofano, Fulvio D’Angelo, Ryan D. Najac, Alberto Picca, Franck Bielle, Anna Luisa Di Stefano, Julie Lerond, Jann N. Sarkaria, Michele Ceccarelli, Marc Sanson, Anna Lasorella, Antonio Iavarone

**Affiliations:** 1 Institute for Cancer Genetics, Columbia University Medical Center, New York, NY USA; 2 Sylvester Comprehensive Cancer Center, University of Miami, Miller School of Medicine, Miami, FL USA; 3 AP-HP, Hôpital de la Pitié-Salpêtrière, Service de Neurologie 2, Paris, France; 4 Sorbonne Université, INSERM Unité 1127, CNRS UMR 7225, Paris Brain Institute, Equipe labellissée LNCC, Paris, France; 5 Department of Neuropathology, Pitié-Salpêtrière–Charles Foix, AP-HP, Paris, France; 6 Department of Neurology, Foch Hospital, Suresnes, Paris, France; 7 Neurosurgery Unit, Spedali Riuniti, Livorno, Italy; 8 Department of Radiation Oncology, Mayo Clinic, Rochester, MN USA; 9 Department of Electrical Engineering and Information Technology (DIETI), University of Naples Federico II, Napoli, Italy; 10 BIOGEM Institute of Molecular Biology and Genetics, Via Camporeale, Ariano Irpino, Italy; 11 Onconeurotek Tumor Bank, Paris Brain Institute ICM, Paris, France; 12 Department of Pathology and Cell Biology, Columbia University Medical Center, New York, NY USA; 13 Department of Pediatrics, Columbia University Medical Center, New York, NY USA; 14 Department of Biochemistry and Molecular Biology, University of Miami, Miller School of Medicine, Miami, FL USA; 15 Department of Neurology, Columbia University Medical Center, New York, NY USA; 16 Department of Neurological Surgery, University of Miami, Miller School of Medicine, Miami, FL USA

**Keywords:** CNS cancer, Machine learning, Cancer genomics, Cancer

## Abstract

Despite producing a panoply of potential cancer-specific targets, the proteogenomic characterization of human tumors has yet to demonstrate value for precision cancer medicine. Integrative multi-omics using a machine-learning network identified master kinases responsible for effecting phenotypic hallmarks of functional glioblastoma subtypes. In subtype-matched patient-derived models, we validated PKCδ and DNA-PK as master kinases of glycolytic/plurimetabolic and proliferative/progenitor subtypes, respectively, and qualified the kinases as potent and actionable glioblastoma subtype-specific therapeutic targets. Glioblastoma subtypes were associated with clinical and radiomics features, orthogonally validated by proteomics, phospho-proteomics, metabolomics, lipidomics and acetylomics analyses, and recapitulated in pediatric glioma, breast and lung squamous cell carcinoma, including subtype specificity of PKCδ and DNA-PK activity. We developed a probabilistic classification tool that performs optimally with RNA from frozen and paraffin-embedded tissues, which can be used to evaluate the association of therapeutic response with glioblastoma subtypes and to inform patient selection in prospective clinical trials.

## Abstract (web summary)

Iavarone and colleagues develop a computational approach called SPINKS to identify master kinases for functional subtypes of human glioblastoma defined using integrated multi-omics data, which show potential as subtype-specific therapeutic targets.

## Main

The classification systems of malignant tumors have evolved in the past 15 years under the pressure of mounting molecular and genetic data and remain an active area of cancer research. The need for more accurate classifiers derives from the urgency of precision oncology and drug development targeting homogeneous tumor subsets^1^. Whereas genomics offers a comprehensive view of the genetic makeup of individual tumors, the integration of genomics, protein profiling and post-translational regulation delivers a deeper understanding of tumor biology and recognizes similarity patterns within individual tumor types, and possibly across multiple types of tumors that can fine-tune targeted therapeutics^2^.

Cancer proteomics consortia have recently provided proteogenomic data and the initial framework for analysis of the proteomic platforms and integration with genomic data^3,4^.

Here, we reconstructed four functional subtypes of glioblastoma (GBM)^5^ using proteomics, phospho-proteomics, acetylomics, metabolomics and lipidomics data using the GBM dataset from the Clinical Proteomic Tumor Analysis Consortium (CPTAC)^6^. We developed a computational approach, Substrate PHosphosite-based Inference for Network of KinaseS (SPHINKS) to generate unbiased kinome-phosphosite networks and extract the master kinases (MKs) driving GBM subtypes. We experimentally validated protein kinase Cδ (PKCδ) and DNA-dependent protein kinase catalytic subunit (DNA-PKcs) as the MKs that sustain cell growth and tumor cell identity of the glycolytic/plurimetabolic (GPM) and proliferative/progenitor (PPR) functional GBM subtypes, respectively. We confirmed PKCδ and DNA-PKcs as MKs in GPM and PPR tumors from pediatric glioma (PG), breast carcinoma (BRCA) and lung squamous cell carcinoma (LSCC) cohorts classified according to the four functional classes that recapitulate metabolic and proliferation tumor cell states. Finally, we developed a probabilistic classification tool for GBM that exhibits optimal performance in both frozen and formalin-fixed, paraffin-embedded (FFPE) tumor tissue for application in cancer clinical pathology.

## Proteogenomic analysis captures functional subtypes of GBM

**Fig. 1:**
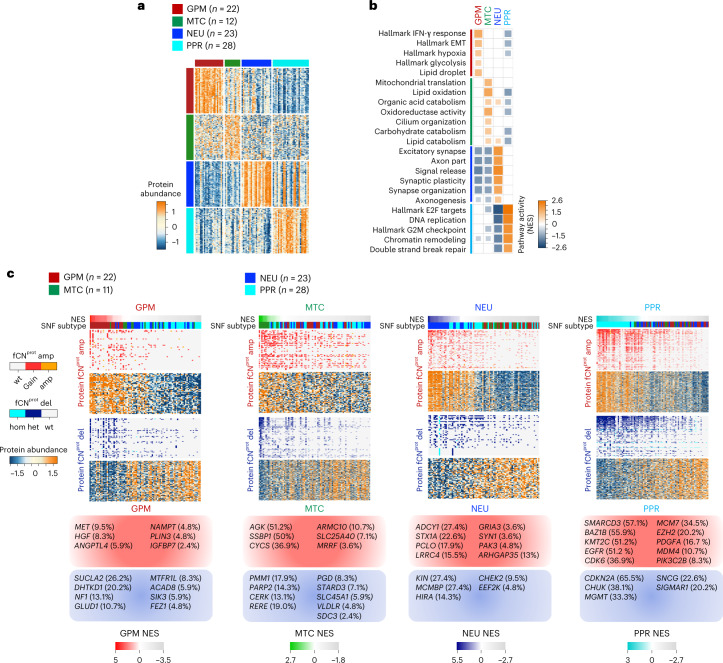
Proteogenomic interpretation of GBM functional subtypes. **a**, Heat map showing the 150 highest scoring proteins in the ranked lists of GPM, MTC, NEU and PPR GBM subtypes (two-sided MWW test). Rows indicate proteins and columns indicate tumors (*n* = 85 GBM samples). Color tracks indicate GBM subtypes (left and top). **b**, Grid plot showing NES of the highest active, non-redundant biological pathways for each GBM subtype (logit(NES) > 0.58, FDR < 0.005; two-sided MWW-GST). The number of GBM samples is as in **a**. IFN, interferon. **c**, Integrative heat map showing CNVs (top) and protein abundance (bottom) of genes with *f*CNV^prot^ gain (amp) or loss (del) (two-sided MWW test). Gains/amplifications are indicated in red; loss/deletions are in blue. In each panel, tumors are ordered from left to right according to highest to lowest subtype activity NES (top track); bottom track indicates tumor classification. The number (*n*) of GBM samples for each subtype is indicated. For each subtype, representative genes with the highest frequency of *f*CNV^prot^ gain (red squares) or loss (blue squares) are listed. wt, wild type; NES, normalized enrichment score; FDR, false discovery rate; GST, gene set test.

**Extended Data Fig. 1:**
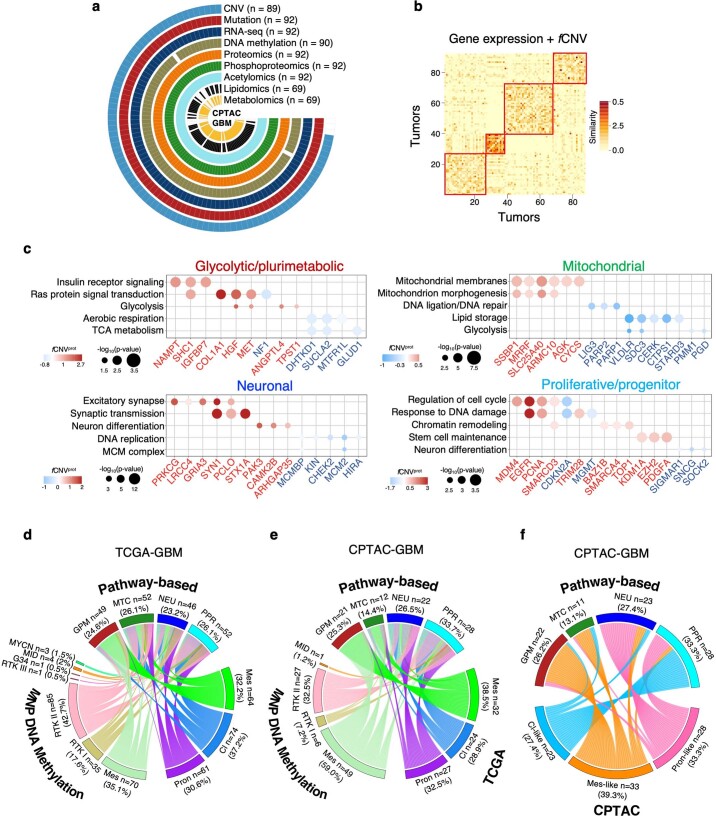
Definition of functional subtypes of GBM by SNF and relationship to prior GBM classifiers. **a**, Circular plot indicating the annotation of data available for each platform and individual tumors of CPTAC-GBM cohort (*n* = 93 GBM samples). The number (*n*) of GBM samples for each platform is indicated. **b**, Integrative clustering of GBM tumors by SNF (*n* = 89 GBM samples). Heat map of patient-to-patient similarity coefficients generated by the integration of subtype-specific gene expression of the highest 50 genes in the ranked lists of the functional subtypes of 52 GBM samples classified as anchors and *f*CNVs associated with the four GBM subtypes from TCGA. Yellow-to-orange scale represents low to high similarity coefficient. **c**, Dot plot showing the genes harboring *f*CNV^prot^ gain or loss and relative pathway enrichment for each GBM subtype (*n* = 85 GBM samples). Dot size indicates significance of the pathway enrichment (*P* < 0.05, Fisher’s exact test) and color the log_2_(FC) of the protein abundance in tumors harboring the *f*CNV^prot^ alteration compared to wild-type tumors (blue to red scale indicate *f*CNV^prot^ gain, red scale; *f*CNV^prot^ loss, blue scale; two-sided MWW test). **d-e**, Chord diagram of GBM subtype assignment of the indicated classifiers in each individual tumor from TCGA (*n* = 199 GBM samples) (d) and CPTAC (*n* = 83 GBM samples) (e) datasets. **f**, Chord diagram of GBM subtype assignment according to the indicated classifiers in each individual tumor from the CPTAC dataset (*n* = 85 GBM samples).

We recently reported a single-cell-guided, pathway-based classification of isocitrate dehydrogenase (IDH) wild-type GBM that consists of four subtypes within two functional branches: neurodevelopment (PPR and neuronal, or NEU) and metabolism (GPM and mitochondrial, or MTC)^5^. Here, we used the proteogenomic data of 92 IDH wild-type GBM from the CPTAC cohort that was profiled by genomics, transcriptomics, proteomics, phospho-proteomics, metabolomics, acetylomics and lipidomics to explore the biology associated with the multi-omics taxonomy and uncover therapeutic opportunities (Extended Data Fig. 1a)^6^. As functional copy-number variations (*f*CNVs), the CNVs of genes associated with coherent transcriptomic changes in *cis* and gene expression were the primary data sources for the pathway-based classifier of GBM^5^, we selected validated *f*CNVs and transcripts as input features of similarity network fusion (SNF)^7^ and obtained four stable clusters (Extended Data Fig. 1b). Using 52 GBM classified according to the highest transcriptomic simplicity score as anchors, we classified 33 of the 40 remaining tumors by the SNF distance matrix (Supplementary Table 1a). Genes differentially expressed by each SNF cluster were enriched with biological activities previously assigned to GPM, MTC, PPR and NEU GBM subtypes (Supplementary Table 2a–c)^5^. Inspection of proteome revealed that the most differentially abundant proteins and enriched pathways coincided with activities biologically congruent with *f*CNV and gene expression-guided functions and recapitulated the predominant biology assigned to each subtype by SNF clustering (Fig. 1a,b and Supplementary Table 2d,e).

To ask whether *f*CNVs impact protein abundance in *cis*, we integrated genomics, transcriptomics and proteomics data to identify genes for which gain or loss correspondingly changed messenger RNA and protein expression (*f*CNV^prot^). Overall, 2,205 genes with *f*CNV gain and 2,837 genes with *f*CNV loss had concordant changes in protein abundance when compared to copy-number neutral samples (Supplementary Table 2f). Among them, 553 (25.08%) *f*CNV^prot^ gains and 415 (14.63%) *f*CNV^prot^ losses segregated with one subtype (Fig. 1c and Supplementary Table 2g–j). *f*CNV^prot^ contributed directly to activation/deactivation of the subtype-specific biological hallmarks (Extended Data Fig. 1c and Supplementary Table 2k).

To understand the relationship between pathway-based classification (GPM, MTC, PPR and NEU) and previously proposed transcriptional (TCGA: proneural, classical and mesenchymal)^8^ and epigenetic (MolecularNeuroPathology (MNP): mesenchymal, RTK I, RTK II, RTK III, MID, MYCN and G34)^9^ subtypes of GBM, we selected 199 and 83 IDH wild-type GBM profiled by both RNA-seq and DNA methylation arrays from TCGA and CPTAC, respectively. We performed a three-way comparison. The GPM subtype exhibited clear association with the mesenchymal subtypes of TCGA and MNP classifiers. Conversely, MTC tumors were mapped to all TCGA and MNP subtypes, with slight preference for RTK II and mesenchymal subtype in the TCGA and CPTAC dataset, respectively (Extended Data Fig. 1d–f and Supplementary Table 1a,b). PPR and NEU had limited overlap with the TCGA and MNP classes, with proneural and RTK I contributing to most PPR and NEU tumors (Extended Data Fig. 1d,e and Supplementary Table 1a,b). Although the epigenetic RTK III, MID, MYCN and G34 subtypes were only minimally represented in TCGA and CPTAC datasets (4.5% and 1.2%, respectively), six of nine tumors were classified as PPR (Extended Data Fig. 1d,e). We also compared functional subtypes with proneural-like, classical-like and mesenchymal-like subtypes reported by CPTAC^6^. GPM tumors were mainly CPTAC mesenchymal-like; however, the mesenchymal-like group also included a significant fraction of MTC cases (Extended Data Fig. 1f), indicating that our classification uniquely discriminates tumors exhibiting alternative metabolic fluxes (MTC and GPM) and clinical characteristics^5^. The CPTAC proneural-like subtype included similar fractions of PPR and NEU, whereas the classical-like subtype was preferentially enriched with PPR tumors.

The analysis confirmed orthogonal distribution of MTC GBM and indicated that, with the description of PPR and NEU subtypes, the pathway-based classifier more accurately captures the neurogenesis stages than the vague definition of proneural state.

## Proteogenomics enables integrative modules of GBM subtypes

**Extended Data Fig. 2:**
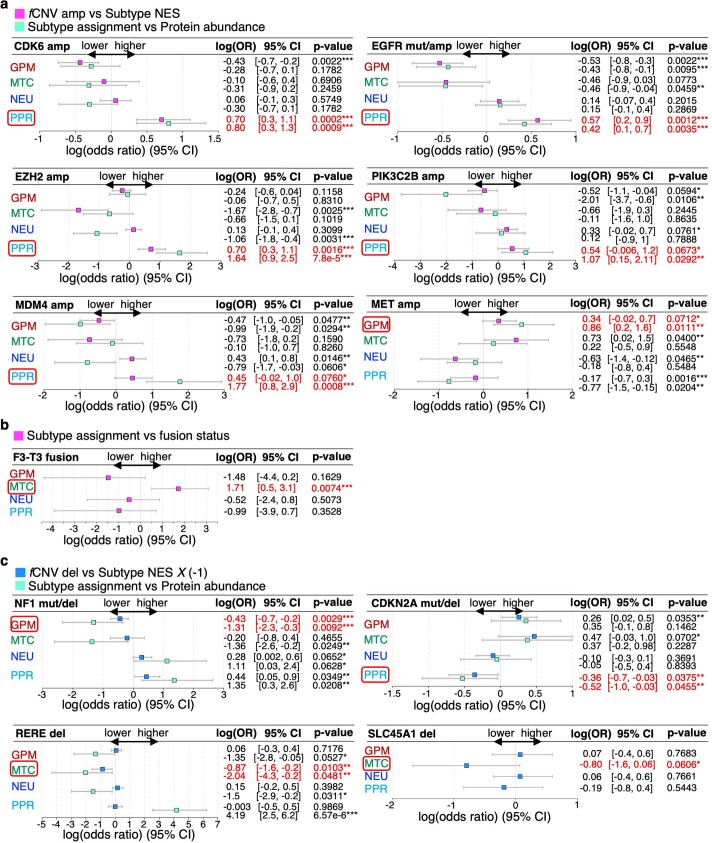
Association between *f*CNV status of GBM driver genes and pathway-based subtypes. **a**, Forest plots showing the association between *f*CNV amplification/mutation status of GBM driver oncogenes and subtype transcriptomic activity (magenta) or abundance of protein of the corresponding gene (light blue) in the CPTAC-GBM cohort (*n* = 84 GBM samples; univariate logistic regression). log(odd ratio) estimates (OR), 95% confidence intervals (CI) and *P* values are reported. log(OR) estimates higher/lower than 0 represent positive/negative association. **b**, FGFR3-TACC3 fusion analysis was performed using a cohort of GBM profiled by FFPE tissue RNA-Seq (*n* = 170 GBM samples; univariate logistic regression). log(OR) estimates, 95% CI and *P* values are reported. **c**, Forest plots showing the association between *f*CNV deletion/mutation status in GBM tumor suppressor genes and subtype transcriptomic activity (blue) or protein abundance of the corresponding gene (light blue; *n* = 84 GBM samples; univariate logistic regression). log(OR) estimates, 95% CI and *P* values are reported. For tumor suppressor genes, subtype activity values (NES) were multiplied by −1 for visualization purposes.

To understand whether each functional subtype of GBM reflects a unique configuration of elements that compose a distinct functional module, from genetic drivers to clinical characteristics such as age, sex and location of the tumor in the brain or radiological features that are obtained at diagnosis by magnetic resonance imaging (MRI), we applied a univariate logistic regression that determined the association of mutations and *f*CNV^5^ with each subtype. In an independent model we asked whether proteins encoded by GBM driver genes provide orthogonal validation to the genetic associations (Extended Data Fig. 2). We found that PPR activity predominantly associated with *f*CNV amplification/mutation/high protein abundance of GBM oncogenes (*CDK6*, *EZH2*, *MDM4* and *EGFR*) and *f*CNV deletion/mutation/protein depletion of *CDKN2A*, all connected to PPR hallmarks. GPM activity was associated with *MET* *f*CNV amplification/high protein abundance and *NF1* *f*CNV deletion/mutation/protein depletion (Extended Data Fig. 2a,c). Confirming our previous findings^10^, the MTC subtype was associated with *FGFR3-TACC3* fusion-positive tumors in the cohort of 178 GBM that we used to validate the probabilistic classifier (see below and Extended Data Fig. 2b)^11^. *f*CNV deletion of *RERE* and *SLC45A1* genes located in the ‘metabolic’ region of chromosome 1p36.23 previously identified as a driver of the MTC subtype^5^ was associated with increased MTC activity. The positive correlation between low RERE protein abundance independently supported the association whereas the SLC45A1 protein was not detected in the CPTAC proteome (Extended Data Fig. 2c). With the limitation of the small number of CPTAC samples, the overall analysis indicated that protein abundance was generally a better indicator of subtype activity than CNV and mutations, a finding that likely reflects control of oncogenic protein abundance by non-genetic factors.

**Fig. 2:**
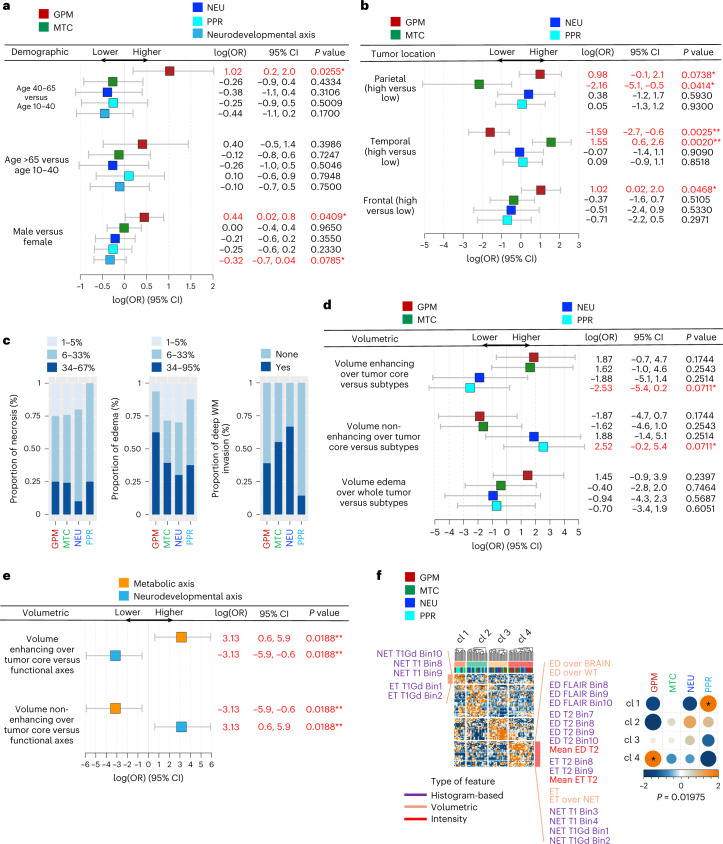
Association between demographic, imaging-based features and functional subtypes. **a**, Forest plots of age and sex association with GBM functional subtypes or the aggregated of PPR and NEU in the TCGA dataset (*n* = 503 GBM samples; univariate logistic regression). log(OR) estimates, 95% confidence intervals (CI) and *P* values are reported (*: *P* < 0.10; **: *P* < 0.05). OR, odds ratio. log(OR) estimates higher/lower than 0 represent positive/negative association. **b**, Forest plots of the association between tumor location and GBM functional subtypes in the TCGA dataset (*n* = 88 GBM samples; univariate logistic regression). log(OR) estimates, 95% CI and *P* values are reported. **c**, Bar plots showing the proportion of necrosis and edema in functional subtypes of GBM from the TCGA cohort (*n* = 63 GBM samples) and deep white matter (WM) invasion from TCGA (*n* = 40 GBM samples) and REMBRANDT (*n* = 14 GBM samples) datasets. **d**, Forest plots of the association between contrast-enhancing, non-contrast-enhancing tumor or edema and GBM functional subtypes in the TCGA dataset (*n* = 88 GBM samples; univariate logistic regression). log(OR) estimates, 95% CI and *P* values are reported. **e**, Forest plot of the association between contrast-enhancing or non-contrast-enhancing tumor and metabolic or neurodevelopmental GBM subtypes in the TCGA dataset (*n* = 88 GBM samples; univariate logistic regression). log(OR) estimates, 95% CI and *P* values are reported. **f**, Unsupervised clustering on 175 differential quantitative radiomic features in GBM subtypes (*n* = 88 GBM samples, left; two-sided MWW test). Top track shows clusters; bottom track shows tumor classification. Representative radiomic features for cluster 1 (enriched with PPR tumors) and cluster 4 (enriched with GPM tumors) are indicated. Association between radiomic clusters and GBM subtypes (right). Circles are color coded and their size reflects the standardized residuals (chi-squared test). Orange-to-blue scale indicates positive to negative enrichment. Asterisks indicates standardized residuals > 1.5.

Next, we analyzed the correlation between clinical characteristics and subtype transcriptomic activity. GPM activity showed significant association with male sex and age between 40 and 65 years. When aggregated, PPR and NEU activities approached significance in association with female sex (Fig. 2a). GPM tumors were more frequently found in the frontal and parietal lobes but were excluded from the temporal region. Conversely, MTC tumors were more frequent in the temporal lobe and were excluded from the parietal lobe, indicating a reciprocal brain location pattern for the metabolic subtypes (Fig. 2b).

To interrogate associations between functional GBM subtypes and radiomic features, we used MRI data available from The Cancer Imaging Archive (TCIA)^12,13^. We categorized the fraction of necrosis, edema and deep white matter invasion and correlated tumor core enhancing and non-enhancing volume and volume of edema with subtype activity (Supplementary Table 1b,c). We also generated an unbiased clustering of histogram-based, volumetric and intensity features. The analyses showed that GPM activity was associated with larger edema and contrast-enhancing volume. PPR activity was associated with greater necrosis, non-enhancing volumes and lower fraction of deep white matter invasion, whereas NEU activity was associated with the lowest volume of necrosis and highest fraction of white matter invasion (Fig. 2c,d). Although the number of samples in each functional subtype was insufficient to provide statistical power, when GPM-MTC or PPR-NEU samples were combined the metabolic subtypes had significantly higher enhancing volume, whereas neurodevelopmental subtypes exhibited larger non-enhancing volumes (Fig. 2e). This scenario was supported by the association of four unsupervised clusters of 175 radiomic features with pathway-based subtypes. Cluster 1 had high non-enhancing and low enhancing volumes as distinctive features and was mostly populated by PPR tumors. Conversely, cluster 4 was enriched with GPM tumors and characterized by overrepresentation of edema and contrast-enhancing volumes but underrepresentation of non-enhancing features (Fig. 2f).

## Multi-omics profiling discriminates functional GBM subtypes

**Extended Data Fig. 3:**
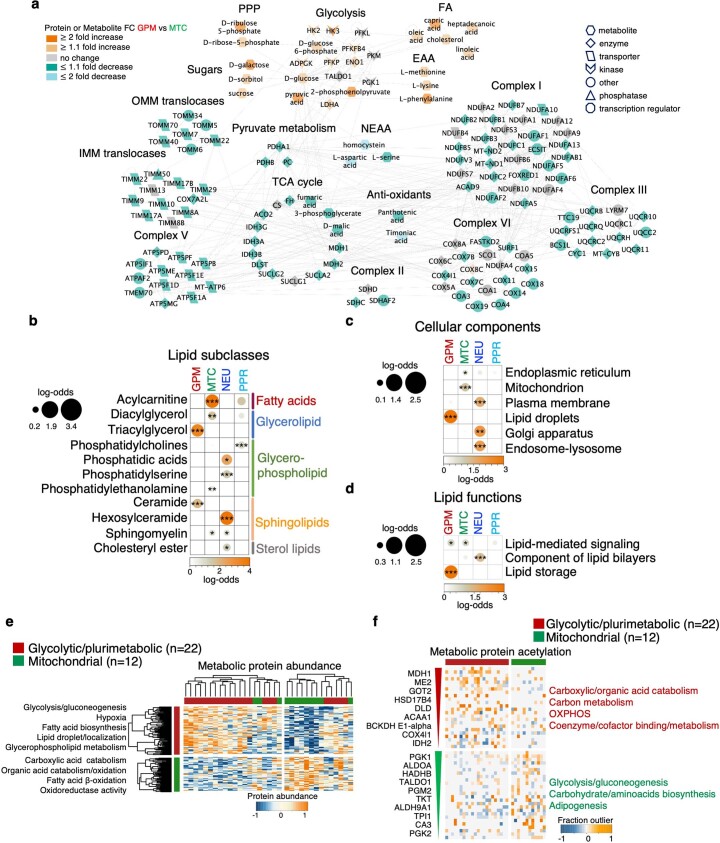
Multiplatform validation of the metabolic axis of the GBM subtypes. **a**, Comparative analysis of the interactome network including intermediate metabolites and enzymes of the indicated metabolic activities in GPM versus MTC tumors (GPM GBM samples: *n* = 16; MTC GBM samples: *n* = 10 for metabolites; GPM GBM samples: *n* = 22; MTC GBM samples: *n* = 12 for proteins; two-sided MWW test). Orange to green scale indicates metabolite/protein increase to decrease in GPM versus MTC samples; [glycolytic intermediates: logit(NES) = 1.76, *P* = 0.0007, mitochondrial intermediates: logit(NES) = −1.65, *P* = 0.018; glycolytic proteins: logit(NES) = 1.27, *P* = 0.017, mitochondrial proteins: logit(NES) = −1.19, *P* = 5.93e-13; two-sided MWW-GST]. **b-d**, Enrichment analysis of **b**, lipid subclasses and **c**, LION terms, grouped according to cellular components and **d**, lipid functions. Lipid subclasses and LION terms significantly enriched in at least one GBM subtype are reported (*n* = 64 GBM samples; log odds ratio > 0, *P* < 0.05; Fisher’s exact test). Circles are color-coded and their size reflect the log odds ratio. Asterisks: * *P* < 0.05, ** *P* < 0.005, *** *P* < 0.001. **e**, Heat map showing unsupervised clustering of metabolic proteins differentially expressed between MTC and GPM samples [log_2_(FC) > 0.3, *P* < 0.05; two-sided MWW test]. Biological pathways significantly enriched in metabolic proteins are reported on the right (log odds ratio > 0, *P* < 0.05; Fisher’s exact test). *n*, number of GBM samples in GPM and MTC subtypes. **f**, Heat map depicting the outlier fraction of acetylated metabolic protein in GPM and MTC tumors (*P* < 0.05; BlackSheep). Representative outlier acetylated proteins are listed on the left according to decreasing *P* value. Biological pathways significantly enriched in outlier acetylated proteins are reported on the right (*P* < 0.0005; Fisher’s exact test). *n*, number of GBM samples in GPM and MTC subtypes.

We inquired whether the divergent features of GPM and MTC subtypes might independently emerge from proteomics, metabolomics and lipidomics platforms. Comparative analysis of GPM and MTC protein profiles showed significantly higher levels of glycolytic enzymes and lower levels of mitochondrial enzymes (translocases, tricarboxylic acid (TCA) cycle and electron transport chain enzymes) in GPM whereas the reciprocal pattern characterized MTC tumors. GPM GBM was preferentially enriched with intermediates of glycolysis, the pentose phosphate shunt, fatty acids, sugars and essential amino acids, whereas MTC GBM contained higher levels of TCA cycle intermediates, antioxidants and non-essential amino acids (Extended Data Fig. 3a).

The analysis of lipidomic data using *LION*^14^ showed that GPM samples had the highest abundance of triacylglycerol, involved in lipid storage and ceramide, which triggers mitochondrial dysfunction (Extended Data Fig. 3b–d and Supplementary Table 2l,m)^15–17^. Conversely, MTC GBM accumulated acyl-carnitine, an integral component of mitochondrial fatty acid oxidation^15^ and diacylglycerol, a lipid second messenger required for membrane fusion and fission^18^. The different lipid composition of GPM and MTC GBM was highlighted by the analysis of lipid cellular components and functions showing enrichment of constituents of lipid droplets in GPM and lipids involved in mitochondrial biogenesis in MTC (Extended Data Fig. 3c,d). Within the neurodevelopmental axis, PPR contained elevated phosphatidylcholines, which are required for cell cycle progression^19^, whereas NEU tumors were enriched in sphingomyelin, phosphatidylserine, hexosyl-ceramide and cholesteryl ester, all essential components of the myelin sheath that surrounds nerve cell axons^20,21^ and phosphatidic acid, a central intermediate for the synthesis of neuronal membrane lipids (Extended Data Fig. 3b–d)^22^.

As lysine acetylation has emerged as a post-translational modification for the regulation of cytoplasmic proteins with crucial metabolic activities and deregulated acetylation of metabolic enzymes can drive metabolic reprogramming of cancer cells^23^, we inquired whether lysine acetylation might differentially regulate metabolism in GPM and MTC subtypes. Unsupervised clustering of metabolism-related proteins differentially expressed between MTC and GPM tumors revealed two clusters, one enriched with GPM tumors and characterized by accumulation of proteins involved in glucose, amino acid and lipid metabolism, and the other enriched with MTC samples and characterized by accumulation of proteins associated with mitochondrial metabolism (Extended Data Fig. 3e and Supplementary Table 3a,b). By applying the outlier enrichment analysis (BlackSheep)^24^ to acetylated proteins, we found that in contrast to global protein abundance, the highest acetylated metabolic proteins in GPM samples included mitochondrial enzymes, whereas MTC samples exhibited hyperacetylation of enzymes implicated in glycolysis and the pentose phosphate pathway as well as amino acid biosynthesis and adipogenesis (Extended Data Fig. 3f and Supplementary Table 3c). As acetylation has been viewed as an inhibitory post-translational modification for the activity of metabolic enzymes^25^, these results present additional levels of coordination of the alternative reprogramming in the metabolic axis of GBM subtypes.

**Fig. 3:**
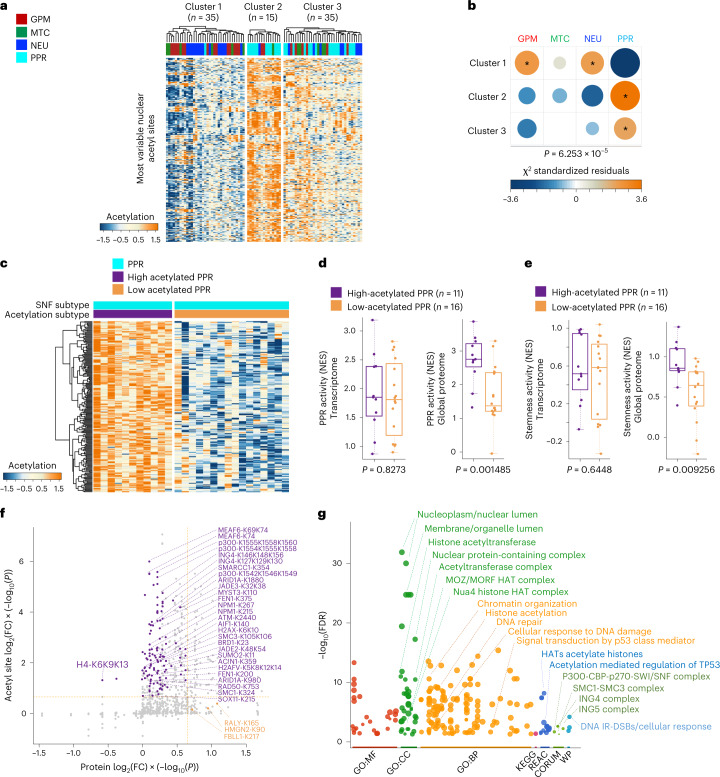
Protein acetylation defines distinct PPR subpopulations. **a**, Heat map showing unsupervised clustering of GBM tumors using the most variable nuclear protein acetyl sites (*n* = 320 acetyl sites). The number (*n*) of GBM samples for each cluster is indicated. **b**, Association between acetylation clusters and functional subtypes of GBM. Circles are color coded and their size reflects the standardized residuals (chi-squared test). Orange-to-blue scale indicates positive to negative enrichment. Asterisks indicate standardized residuals > 2. The number (*n*) of GBM samples is as in **a**. **c**, Heat map showing unsupervised clustering of differential acetylated nuclear proteins in PPR tumors with high (*n* = 11 PPR GBM samples in cluster 2 of **a**) and low (*n* = 16 PPR GBM samples in cluster 3 of **a**) acetylation of nuclear proteins (log_2_(FC) > 0.3, *P* < 0.001; two-sided MWW test). **d**, Box plots of PPR activity calculated from the transcriptome (left) or global proteome (right) in PPR GBM with low and high acetylation (two-sided MWW test). Box plots span the first to third quartiles and whiskers show 1.5× interquartile range. The number (*n*) of PPR GBM samples with low and high acetylation is indicated. **e**, Box plots of stemness activity calculated from transcriptome (left) or global proteome (right) in PPR GBM with low and high acetylation (two-sided MWW test). Box plots span the first to third quartiles and whiskers show 1.5× interquartile range. The number (*n*) of PPR GBM samples with low and high acetylation is indicated. **f**, Starburst plot integrating global protein and acetyl site abundance of high- (*n* = 11 PPR GBM samples) versus low-acetylated PPR GBM (*n* = 16 PPR GBM samples; two-sided MWW test). The *x* axis indicates protein log_2_(FC) multiplied by −log_10_(*P*). The *y* axis indicates acet*y*l site log_2_(FC) multiplied by −log_10_(*P*). The horizontal and vertical lines denote the cutoff of log_2_(FC) = 0.5 multiplied by −log_10_(*P* = 0.05). **g**, Gene Ontology overrepresentation analysis of acetylated proteins in **f** using gProfiler (FDR < 0.05). The number (*n*) of PPR GBM samples with low and high acetylation is as in **f**. FC, fold change.

We then examined the pattern of nuclear protein acetylation across GBM subtypes. Unsupervised clustering of the most variable nuclear protein acetylation sites uncovered three clusters (Fig. 3a). Cluster 1 was acetylation cold and enriched in GPM and NEU tumors. Cluster 2 included tumors with the highest acetylation and was almost exclusively composed of PPR samples. Cluster 3 was an intermediate/low-acetylation cluster that included 46% of PPR samples (16 tumors) intermixed with GPM, NEU and MTC tumors (Fig. 3b). Thus, the PPR subtype seems to be divided into two subgroups, exhibiting high and low nuclear protein acetylation, respectively (Fig. 3c and Supplementary Table 3d). Tumors in the high-acetylation PPR subcluster had the highest proteomics but not transcriptomics proliferation/stemness scores, thus highlighting the specific role of the post-translation acetyl modification in this subtype (Fig. 3d,e). Differential acetylation of PPR GBM among high-acetylation and low-acetylation subclusters involved specific acetylation sites of histone and non-histone acetyltransferases (lysine acetyltransferases, KATs) whose enzymatic activity is activated by auto-acetylation^26,27^. Such activation was clearly manifested in high-acetylation PPR by the elevated level of acetyl-lysines in the HAT domain of p300 (K1554, K1555, K1558 and K1560) and functionally similar residues in the HAT domain of other KATs such as members of the MYST complexes (MEAF6, ING4, JADE2, JADE3 and MYST3; Fig. 3f and Supplementary Table 3e). The latter introduce acetylated marks upon histones H2, H3 and H4 (ref. ^28^), which were recovered as hyperacetylated (H2AX, H2AFV and HIST2H4B) in high-acetylation PPR. Besides KATs and histones, chromatin-modifying enzymes and enzymes involved in DNA damage response (DDR) and DNA replication stress (RS) were hyperacetylated in high-acetylation PPR, suggesting that acetylation contributes to the activation of these biological functions in PPR GBM (Fig. 3g).

## Sustained RS and DDR signaling characterizes PPR GBM

**Fig. 4:**
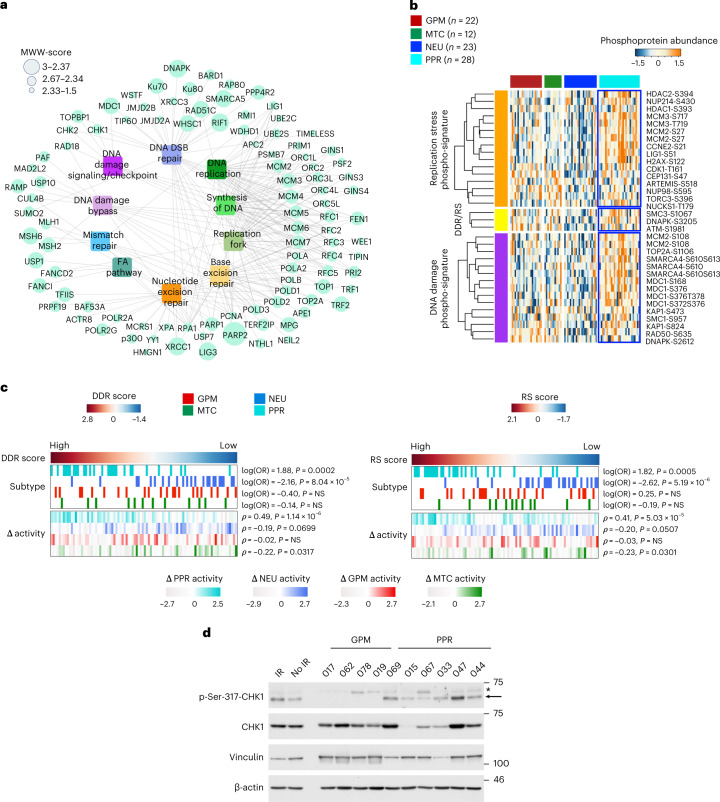
GBM of the PPR subtype exhibits phospho-programs of DDR activity and replication stress and distinct sensitivity to DDR inhibition. **a**, DDR signaling network including the most enriched pathways and the highest abundant proteins in PPR GBM (MWW score > 1.5) compared to the other subtypes (logit(NES) > 1, *P* < 0.001, two-sided MWW-GST, *n* = 85 GBM samples). FA, Fanconi anemia. **b**, Heat map showing the phospho-protein abundance of biologically validated phosphorylation sites upregulated by irradiation-induced DDR and aphidicolin-induced DNA RS. The number (*n*) of GBM samples for each subtype is indicated. **c**, DDR (left) and RS-induced (right) signature score of GBM classified according to four functional subtypes. Top track, left to right represents tumors ranked by the highest to the lowest DDR or RS score. Heat map showing tumor subtype assignment (Fisher’s exact test) (top). Each row represents a functional subtype. Heat map showing for each tumor the difference between subtype-specific proteomic and transcriptomic activity (Spearman’s correlation) (bottom). Each row represents a subtype-specific activity. White to red, GPM; green, MTC; blue, NEU; cyan, PPR. Subtype-specific color scale indicates lowest to highest Δ enrichment score for each subtype. The number (*n*) of GBM samples is as in **b**. **d**, Immunoblot of GPM PDOs (*n* = 4 PDOs, each derived from an independent patient) and PPR PDOs (*n* = 6 PDOs, each derived from an independent patient) analyzed using the indicated antibodies. Vinculin and β-actin are shown as loading control. * indicates nonspecific band. The experiment was repeated twice with similar results. NS, not significant. Source data

The proteomic profiling of PPR GBM combined molecular marks of proliferation with activation of DDR (Fig. 1b). Moreover, PPR tumors exhibited overrepresentation of DNA replication/replication fork and DNA double-strand break repair (DDSB) proteins, suggesting that enhanced RS may promote DDR signaling (Fig. 4a). To test this hypothesis, we performed data mining and ontology integration from mass-spectrometry datasets to identify phosphosites increased in cells treated with irradiation, which causes DDSB lesions, ATR inhibitors or hydroxyurea that induce RS (Methods). We selected 15 and 16 experimentally validated phosphosites specific for cells undergoing DDSB and RS, respectively and 3 phosphosites common to DDSB and RS. Compared to other tumor subtypes, PPR contained elevated levels of 11 (73.3%) and 10 (62.5%) of DDR and RS signature phosphosites, respectively (Fig. 4b and Supplementary Table 4). Using DDR and RS phospho-proteomic signatures, we computed DDR and RS enrichment scores for each tumor and found higher scores in PPR than other subtypes, with the NEU group characterized by the lowest scores (Fig. 4c, top). The highest PPR scores were retained even when tumors were classified according to the difference between proteomic and transcriptomic subtype activity (Fig. 4c, bottom), thus reinforcing the significance of the proteome for the association between DDR/RS and PPR subtype. Western blot using CHK1-ser-317 phosphorylation as a basal DDR biomarker of ATR-activated CHK1 (ref. ^29^) showed that GBM patient-derived organoids (PDOs) classified as PPR^5^ exhibited higher levels of basal DDR/RS than GPM PDOs (Fig. 4d).

## Master kinase analysis uncovers GBM subtype-specific kinases and actionable dependencies

**Fig. 5:**
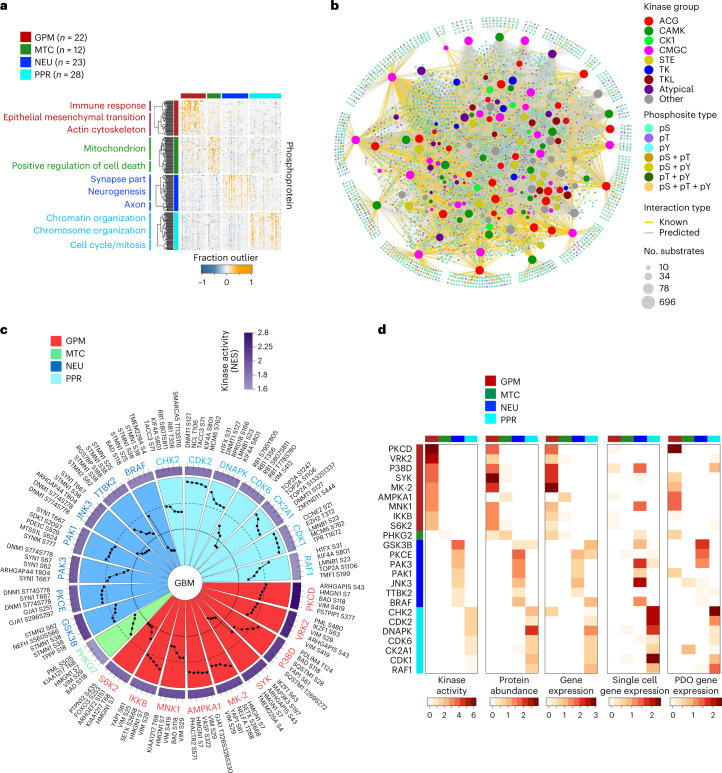
Protein phosphorylation-kinase networks by SPHINKS reveal subtype-specific master kinases and signaling. **a**, Heat map depicting the 70 highest significant outlier phosphorylated proteins in each functional GBM subtype (*P* < 0.005; BlackSheep). Unsupervised clustering and biological pathways significantly enriched are presented on the left (*P* < 0.01; Fisher’s exact test). The number (*n*) of GBM samples for each subtype is indicated. **b**, Global kinase–substrate phosphosite interactome inferred by SPHINKS. Nodes represent kinases and substrate phosphosites and lines their interactions. Kinase families and phosphorylated amino acid residues are indicated by different colors. Node size of the kinases is proportional to the number of interacting phosphosites. Yellow interactions indicate substrate phosphosites reported in the PhosphoSitePlus database; gray interactions are inferred new interactions. The number (*n*) of GBM samples is as in **a**. **c**, Circular plot depicting the most active kinases in each GBM subtype compared to all other subtypes (effect size > 0.3, *P* < 0.01; two-sided MWW test) with the outermost circle representing the color scale of kinase activity. The five predicted kinase-regulated phosphorylation sites with the highest SPHINKS score are indicated by black dots with SPHINKS score within the dashed line, > 0.95; SPHINKS score between dashed and continuous line, 0.95–0.90; and SPHINKS score inside the continuous line, < 0.90. The number (*n*) of GBM samples is as in **a**. **d**, Heat maps showing kinase activity (NES), MWW protein abundance score and MWW gene expression score of SPHINKS MKs specific for each CPTAC-GBM subtype (two-sided MWW test, *n* = 85 GBM samples). Heat maps depicting MWW gene expression score of the same kinases in single GBM cells (*n* = 17,367 single glioma cells) and PDOs (*n* = 79 PDOs) signify the cancer cell intrinsic expression of the top-scoring kinases identified by SPHINKS. Only values of logit(NES) > 0.58 are shown.

**Extended Data Fig. 4:**
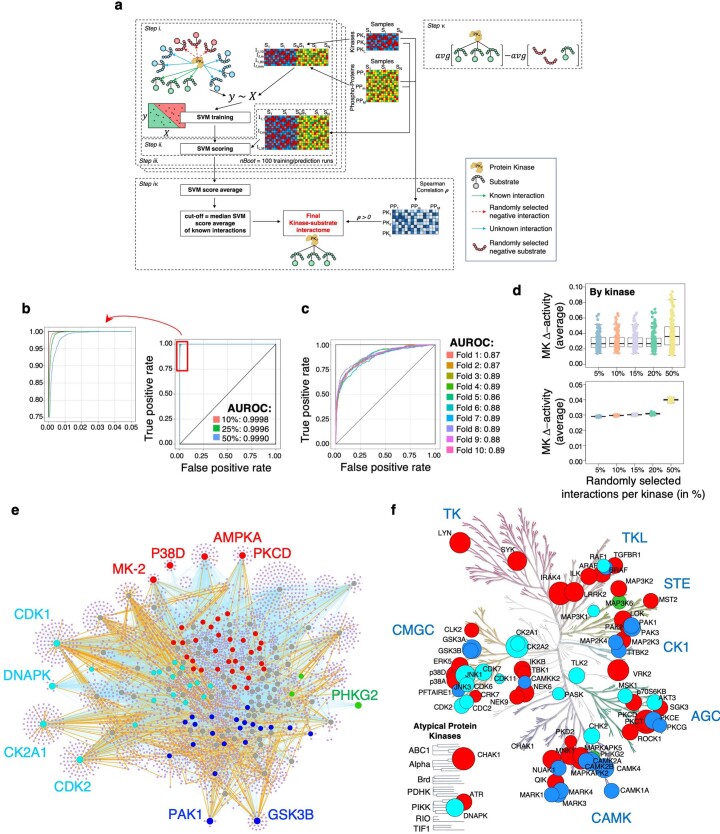
Computational strategy for the identification of MKs in functional GBM subtypes and benchmarking of SPHINKS approach. **a**, The reconstruction of an unbiased kinome network combines SVM classifiers trained on different instances of the negative set as follows: (step i) train SVM classifier on validated kinase-substrate interactions (green arrows, positive training set) and a subset of randomly selected unknown interactions (red dotted arrow, negative set) using kinase abundance from proteomics and substrate abundance from phosho-proteomics; (step ii) compute a score for all the interactions in the network according to the SVM classifier; (step iii) perform bagging and obtain the average SVM scores; (step iv) retain only interactions whose average score was above the average SVM score threshold (50% of the known interactions) and whose Spearman’s correlation was positive; (step v) calculate MKs activity as the difference of two terms, the weighted average of the predicted substrate’s abundances using the SPHINKS score as weight (left), and the weighted average of randomly selected control substrate-set (right). **b**, ROC curves of the predictions of the interactions by SPHINKS derived from simulated phospho-proteomic matrix with different rates of missing values. The top-left side of plot was magnified for accurate visualization. **c**, ROC curves of the interactions by SPHINKS for each of the 10 cross-validation iterations of experimentally validated interactions. **d**, Box plots of the average kinase Δ-activity (percentage) from unperturbed versus 100 networks perturbed with random phosphosites interactions for each kinase replacing true interactions in the network (*p* = 5%, 10%, 15%, 20%, 50%). In the upper plot, each dot represents the average Δ-activity for each kinase across all runs at each perturbation percentage; in the lower plot, each dot represents the average Δ-activity for each run across all kinases at each ratio of perturbation. Box plots span the first to third quartiles and whiskers show the 1.5 × interquartile range. **e**, Kinase-substrate interactome from SPHINKS highlighting MKs for each functional subtype indicated by colors: red, green, blue and cyan, MKs in GPM, MTC, NEU, and PPR, respectively (effect size > 0.3, *P* < 0.01; two-sided MWW test; *n* = 85 GBM samples). Nodes represent kinases and substrates, and lines their interactions. Gray nodes are subtype non-specific kinases; purple nodes are kinase-targeted phosphosites substrates. Orange lines indicate kinase-phosphosite interactions from PhosphoSitePlus; cyan lines represent novel kinase-substrate interactions inferred by the SPHINKS. **f**, MKs significantly active in each functional GBM subtype were mapped onto the human kinome tree. Red, green, blue and cyan, MKs in GPM, MTC, NEU, and PPR, respectively. The size of the circles is proportional to the kinase activity. The number of GBM samples is as in e.

To begin exploring the phospho-proteomics landscape of GBM subtypes and their organization, we cataloged phosphosites specific for each GBM subtype and applied the outlier enrichment analysis. We obtained four phosphosite modules of overrepresented pathways that summarized previously assigned subtype hallmarks (Fig. 5a and Supplementary Table 5a–c). We then sought to link phosphosite enrichment to the activity of GBM subtype-specific protein kinases. To this aim, we developed SPHINKS, which integrates proteomics and phospho-proteomics profiles to build an interactome of kinase–phospho-substrate pairs that are scored according to the strength of their interaction across all samples (Fig. 5b). The GBM-specific kinase–phosphosite interaction network was generated using a semi-supervised support vector machine (SVM) algorithm trained on experimentally validated kinase–substrate phosphosite pairs from the PhosphoSitePlus database^30^. SPHINKS produced a GBM kinase–phosphosite interactome comprising 13,866 predicted interactions between 154 kinases and 3,186 phospho-substrates (Extended Data Fig. 4a(i–iv)). To benchmark SPHINKS, we assessed the impact of missing data in the kinase–phosphosite interactome by comparing networks reconstructed from the CPTAC-GBM un-imputed matrix of phosphosites lacking missing values (gold standard, 7,302 phosphosites) and controlled simulations of imputed matrices composed of different ratios of phosphosite missing values (Methods). Receiver operating characteristics (ROC) analysis showed that regardless of the different thresholds of missing values, the area under the curve (AUC) was consistently close to 1, indicating that the output of SPHINKS was not affected by missing values (Extended Data Fig. 4b). To evaluate the accuracy of SPHINKS to correctly predict kinase–phospho-substrates, we performed a tenfold cross-validation by randomly dividing validated interactions into ten subsets for training and testing. AUC values of all iterations between 0.86–0.89 indicated high prediction accuracy (Extended Data Fig. 4c). As some of the selected phosphosites in the negative test set might be true substrates, AUC values are likely to be underestimated. To test the stability of SPHINKS kinase activity estimates, we generated 100 independent networks for each kinase and perturbed them by replacing a predetermined percentage of phospho-substrates with random phosphosites. Average Δ activity scores (difference between unperturbed and perturbed networks) indicated a remarkable stability of the kinase activity estimate inferred by SPHINKS (median Δ activity = 3%, for perturbations ≤20% interactions in both analyses; median Δ activity = 4% in both analyses, maximum of 10% in kinase analysis, for perturbations of 50%; Extended Data Fig. 4d).

**Extended Data Fig. 5:**
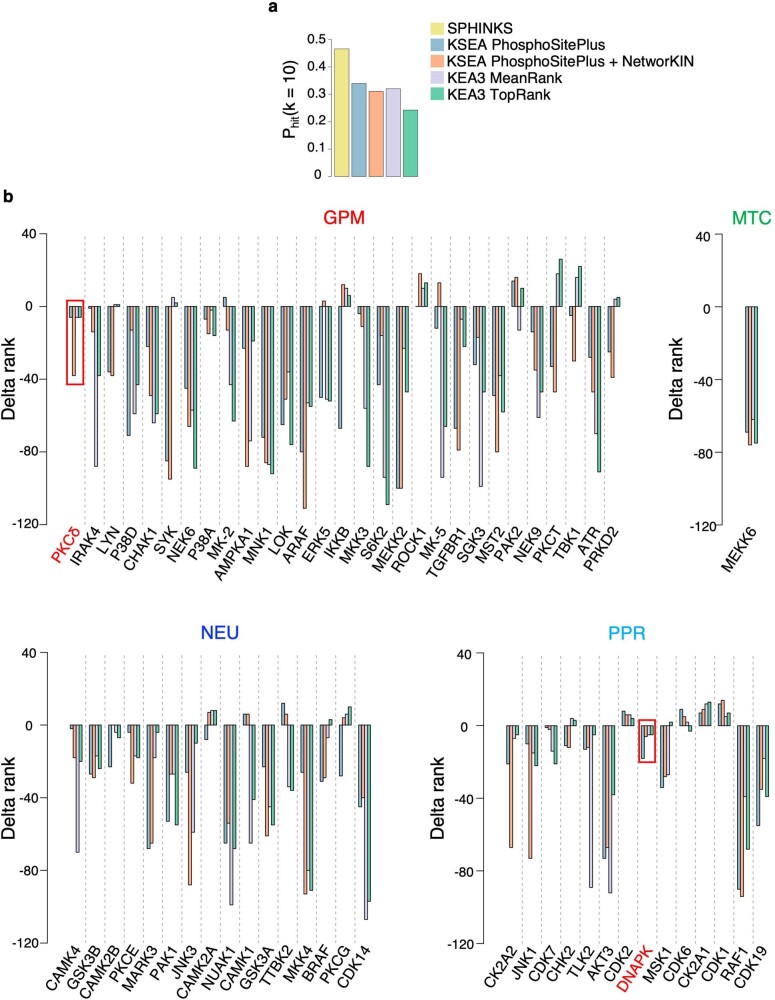
Benchmarking of SPHINKS against previously published kinase-substrate inference methods. **a**, Bar plot showing the probability of correctly identifying upregulated or downregulated kinases by the analysis of the ‘top-10-hit’ using the indicated inference methods (*n* = 103 kinase perturbations). **b**, Bar plot of the differential rank (Δ-rank) of activity between SPHINKS and the indicated inference methods for the kinases significantly active in each GBM subtype by SPHINKS and common to the networks of all five approaches (*n* = 85 GBM samples). Kinases are ordered according to the rank of activity by SPHINKS.

To uncover MKs associated with distinct GBM subtypes, we implemented single-sample MK analysis by computing the weighted strengths of connectivity between kinase and predicted substrate phosphosites against random phosphosites for each tumor and testing the contribution of each MK in each subtype by Mann–Whitney–Wilcoxon (MWW) test^10^ (Extended Data Fig. 4a(v) and Supplementary Table 5d). GPM, PPR and NEU GBM exhibited rich and interconnected kinase–substrate networks as opposed to the MTC subtype that was sustained by a more limited network (Fig. 5c and Extended Data Fig. 4e). Mapping the predicted subtype-specific MKs onto the human kinome tree showed a random distribution across kinase families (Extended Data Fig. 4f). We validated subtype-specific MKs in bulk GBM, single-cell RNA-seq (scRNA-seq) data from 17,367 GBM cells and 79 GBM PDOs^5^. mRNA and protein of the kinases identified by SPHINKS-MK were generally upregulated in bulk tumors and cells of the corresponding subtype (Fig. 5d and Supplementary Table 5e). We compared SPHINKS-MK with kinase–substrate enrichment analysis (KSEA)^31^ and kinase enrichment analysis 3 (KEA3)^32^. Unlike SPHINKS that reconstructs context-specific kinase–phospho-substrate networks and detects potentially new kinase–substrate interactions, KSEA and KEA3 derive kinase activity from networks of experimentally validated phospho-substrates. For KSEA, we obtained kinase activities from validated interactions from PhosphoSitePlus (KSEA PhosphoSitePlus) or predicted relationship from NetworKIN (KSEA PhosphoSitePlus + NetworKIN). For KEA3, we applied MeanRank and TopRank for ranking the integrated kinase activity from 11 protein–protein and kinase–substrate interaction libraries. We used a dataset reporting changes in the abundance of phospho-proteins after perturbation of upstream kinases^33,34^ (103 kinase perturbation for 30 kinases and 61,181 phosphosites, the ‘gold standard’) and the metric defined as ‘top-k-hit’, which focuses on the top kinase predictions^34^. SPHINKS produced higher activity scores than other methods and was superior in correctly identifying the perturbed kinases (Extended Data Fig. 5a). We also calculated the difference between the activity rank inferred by SPHINKS and each of the other methods (Δ rank score) of 129 kinases common to all five methods for each GBM subtype using CPTAC-GBM proteomic/phospho-proteomic data. For all comparisons, most of the kinases exhibited a negative Δ rank score, indicating that SPHINKS has a consistently higher predictive power than other approaches (Extended Data Fig. 5b).

## PKCδ and DNA-PKcs are subtype-specific actionable MKs in GPM and PPR

**Fig. 6:**
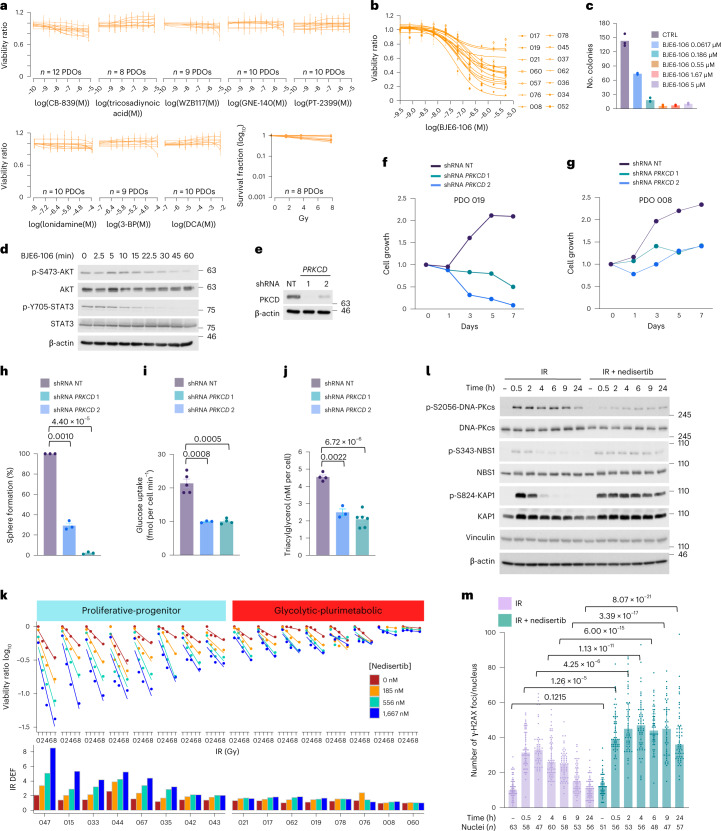
Validation of dependency of GBM cells on specialized protein kinases. **a**, Viability curves of PDOs, each derived from an independent patient. Each curve represents one independent PDO assayed for the indicated compound or IR. Data in each curve are mean ± s.d. of *n* = 3 or 6 technical replicates for compound treatment (Source Data Fig. 6) and *n* = 8 technical replicates for IR. Experiments were performed twice with similar results. **b**, Viability curves of GPM PDOs (*n* = 14 PDOs, each derived from an independent patient) treated with BJE6-106. Data in each curve are mean ± s.d. of *n* = 6 or 18 technical replicates for each PDO (Source Data Fig. 6). The experiment was repeated three times with similar results. **c**, Colony-forming assay using GPM PDO cells treated with BJE6-106. Data are the mean of *n* = 3 technical replicates from one representative experiment. Experiment was repeated twice with similar results. CTRL, control. **d**, Western blot of GPM PDO cells treated with 50 μM of BJE6-106. Experiment was repeated twice with similar results. **e**, Western blot of GPM PDO cells transduced with lentivirus expressing two independent shRNAs targeting *PRKCD* or non-targeting shRNA (NT). Experiment was repeated three times with similar results. **f**,**g**, Growth curves of two independent GPM PDOs, PDO 019 (**f**) and PDO 008 (**g**) transduced as in **e**. Data are mean of *n* = 5 (**f**) and *n* = 6 (**g**) technical replicates from one representative experiment. Experiments were repeated twice with similar results. **h**, Quantification of sphere-forming assay for GPM PDO cells (PDO 008) transduced as in **e**. Data are mean ± s.d. of *n* = 3 independent infections/biological replicates. **i**, Rate of glucose uptake in GPM PDO cells (PDO 019) transduced as in **e**. Data are mean ± s.d. of *n* = 6 for shRNA NT, *n* = 3 for shPRKCD 1 and *n* = 4 for shPRKCD 2 technical replicates from two independent infections/biological replicates. **j**, Concentration of triacylglycerol in GPM PDO cells (PDO 019) transduced as in **e**. Data are mean ± s.d. of *n* = 4 for shRNA NT, *n* = 3 for shPRKCD 1 and *n* = 6 for shPRKCD 2 technical replicates from two independent infections/biological replicates. **k**, Cell viability after IR minus or plus nedisertib of PPR PDOs (*n* = 8 PDOs, each derived from an independent patient) and GPM PDOs (*n* = 8 PDOs, each derived from an independent patient). Data in each curve are mean of *n* = 4 technical replicates. Experiment was repeated twice with similar results. **l**, Western blot of PPR PDO cells treated with IR (4 Gy) or IR plus nedisertib (556 nM). Experiment was repeated twice with similar results. **m**, Quantification of γ-H2AX foci per nucleus in PPR PDO cells (PDO 044) after treatment as in **l**; the number (*n*) of nuclei is indicated (Source Data Fig. 6). Data are mean ± s.e.m. In each quantitative experiment, significance was established by two-tailed *t*-test, unequal variance or the Mann–Whitney test for experiment in **m**. In western blots, vinculin and β-actin are shown as loading controls. Source data

**Extended Data Fig. 6:**
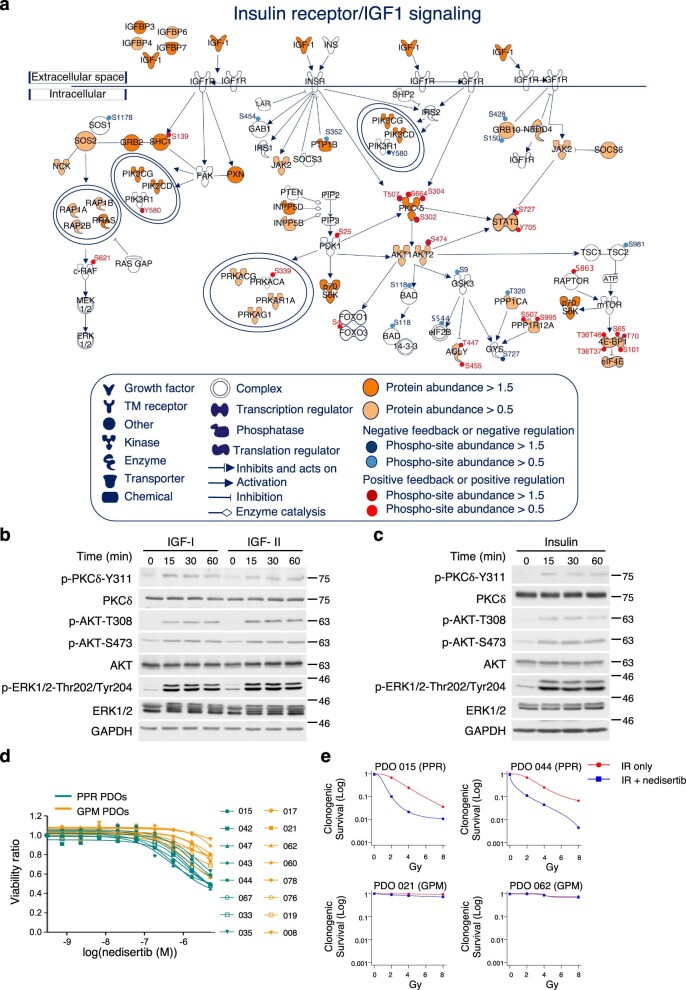
Global and phospho-proteomics events in insulin receptor/IGF-PKCδ pathway in GPM GBM and enrichment of DDR and RS phospho-proteins as a specific feature of PPR GBM. **a**, Signaling network highlighting the molecules and proteins involved in IGF-I/insulin signaling of GPM GBM tumors. Orange or red scale indicates the MWW score derived from the proteomic or phosphosite ranked list of GPM tumors when compared to the others, respectively (two-sided MWW test, *n* = 85 GBM samples). Molecules in white are proteins not profiled or whose abundance was not significantly higher in GPM when compared to the other subtypes. **b-c**, Western blot analysis of GPM PDO cells incubated with b, IGF-I (10 ng/ml), IGF-II (10 ng/ml) and c, insulin (100 ng/ml) for the indicated times using the indicated antibodies. GAPDH is shown as a loading control. Each experiment was repeated independently 2 times with similar results. **d**, Viability curves of *n* = 8 PPR PDOs each derived from an independent patient and *n* = 8 GPM PDOs, each derived from an independent patient treated with increasing concentration of Nedisertib. Data are mean ± s.d. of *n* = 4 technical replicates for each PDO from one representative experiment. Experiments were repeated 2 times with similar results. **e**, Quantification of clonogenic assay of 2 PPR PDOs (PDO 015 and PDO 044, top panels) each derived from an independent patient and 2 GPM PDOs (PDO 021 and PDO 062, bottom panels) each derived from an independent patient treated with IR or IR plus Nedisertib (1667nM). Data are mean of *n* = 3 technical replicates from one representative experiment. Experiments were repeated 2 times with similar results. Source data

The application of SPHINKS-MK uncovered PKCδ as the top-scoring MK of the GPM subtype (Fig. 5c). PKCδ controls crucial steps of glucose and lipid metabolism in multiple tissues^35^. In cancer, PKCδ is a central signaling node of the insulin–IGF–AKT–mTOR pathway that orchestrates metabolic reprogramming toward aerobic glycolysis and increased uptake of nutrients^36–38^. PKCδ also mediates resistance to antitumor therapies possibly by upregulating glucose uptake in cancer cells^39^. As the metabolic functions controlled by PKCδ are hallmarks of GPM GBM^5^, we tested the role of PKCδ in the plurimetabolic phenotype and viability of this subtype. Exposure of GBM PDOs classified as GPM to eight compounds targeting different glycolytic enzymes or irradiation confirmed that each treatment was ineffective in these cells (Fig. 6a). Next, we asked whether activation of PKCδ in GPM GBM segregated with insulin-IGF-AKT signaling. By the comparative analysis of protein and phospho-protein abundance of pathway-specific signaling molecules in GPM versus all other subtypes, we found that crucial components of the insulin–IGF–AKT pathway were activated in GPM tumors by elevation of protein abundance and/or phosphorylation, and co-segregated with PKCδ abundance and activation (Extended Data Fig. 6a). AKT1/2 and STAT3, central nodes in insulin–IGF-PKCδ signaling, were activated in GPM GBM. Additionally, activation of the mTOR kinase (RAPTOR-ser-863) and substrates (p70S6K and 4E-BP-ser-37/thr-46 phosphorylation) was consistent with the relevance of this pathway for the metabolic reprogramming of GPM tumors (Extended Data Fig. 6a). Stimulation of GPM PDOs by IGF1/2 and insulin induced phosphorylation of PKCδ on tyr-311, a phosphosite crucial for its activity^40^, concurrently with AKT-thr-308 and ser-473 phosphorylation (Extended Data Fig. 6b,c). To test the essentiality of PKCδ for fitness and the plurimetabolic state of GPM cells, we treated GPM PDOs with BJE6-106 (ref. ^41^), a third-generation inhibitor of PKCδ and found that most of the tested models exhibited marked sensitivity to PKCδ inhibition (Fig. 6b). BJE6-106 also caused dose-dependent inhibition of colony formation (Fig. 6c) and time-dependent decrease of AKT-ser-473 and STAT3-tyr-705 phosphorylation (Fig. 6d). Genetic knockdown of the *PRKCD* gene (Fig. 6e) corroborated the requirement of PKCδ for growth and viability of GPM PDOs (Fig. 6f–h) as well as glucose uptake and lipid accumulation (Fig. 6i,j).

The catalytic subunit of DNA-dependent protein kinase (DNA-PKcs) was among the most active MK in the PPR subtype of GBM (Fig. 5c,d). DNA-PKcs is one of the three members of PIKKs with principal roles in the activation of DDR. DNA-PKcs is activated by multiple types of genotoxic stress, including DDSB and RS^42,43^. Given the specific activation of DDR and RS in PPR GBM (Figs. 1b, 3g and 4), we postulated that active DNA-PKcs may counter the increased rates of DNA replication and DDR in PPR cells. Consequently, we asked whether inhibition of DNA-PKcs with M3814 (nedisertib), a DNA-PKcs inhibitor currently in clinical studies^44^, promotes vulnerability of PPR GBM when used in combination with ionizing radiation (IR), the key element in the standard of care for patients with GBM. Treatment of PPR GBM PDOs with a nedisertib–IR combination markedly reduced tumor cell viability compared to each individual treatment, with a radiation dose enhancement factor (DEF) > 2 for six PPR PDOs. Conversely, nedisertib–IR combination was ineffective in GPM PDOs (Fig. 6k and Extended Data Fig. 6d). We confirmed these results using the clonogenic assay as a quantitative method of radiosensitivity (Extended Data Fig. 6e). Exposure of PPR PDOs to IR rapidly induced phosphorylation of DNA-PKcs ser-2056, the key autophosphorylation site marking kinase activation^45^. As expected, nedisertib inhibited ser-2056 phosphorylation in irradiated cells (Fig. 6l). Combinatorial treatment caused persistent DNA damage as shown by sustained phosphorylation of ser-343 of NBS1 and ser-824 of KAP1, indicators of active DDSB, as opposed to rapid de-phosphorylation in PDOs exposed to IR alone (Fig. 6l). Consistently, the number of γ-H2AX foci, which regressed to basal levels in PPR cells treated with irradiation alone, remained elevated throughout the course of the experiment in the presence of DNA-PKcs inhibition (Fig. 6m).

## Functionally conserved pediatric and adult cancer subtypes share MKs

In an effort to ascertain whether the key biological functions discriminating the GBM subtypes coalesce into grouping patterns sharing the same kinase-driven dependencies, we first determined whether a functional classification could be obtained in PG, BRCA and LSCC for which genomics, proteomics and phospho-proteomics datasets are available^46–48^.

**Fig. 7:**
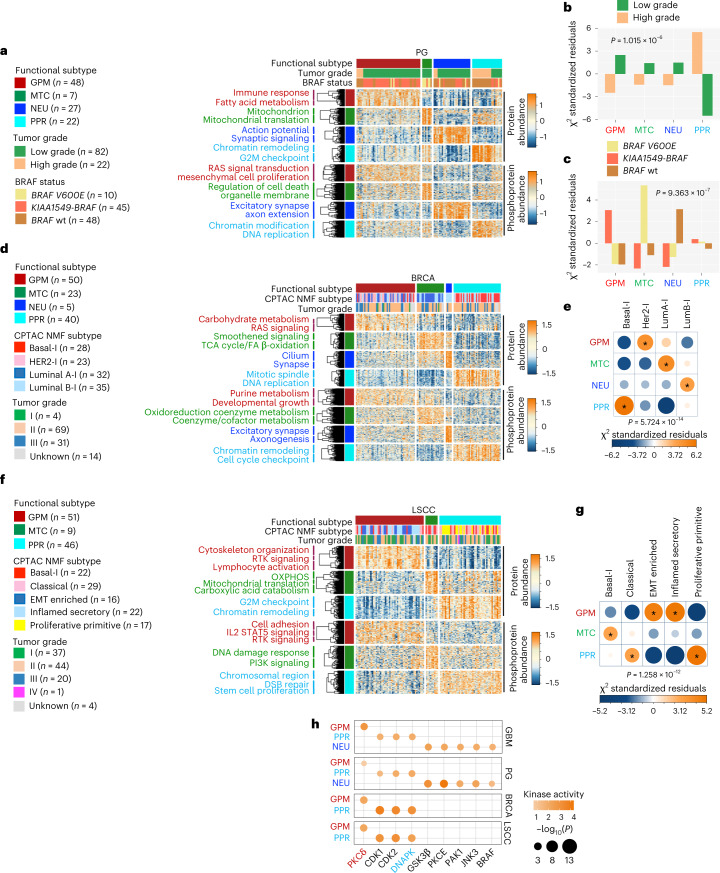
Functional activities of GBM subgroups classify different cancer types and inform survival and master kinases. **a**, Heat map showing the 150 highest scoring proteins (top) and phosphosites (bottom) of four functional subtypes of CPTAC-PG; rows show proteins/phosphosites and columns show tumors (*n* = 104 PG samples; two-sided MWW test). Left and top tracks indicate the functional subtypes; middle track indicates tumor grade; and bottom track indicates *BRAF* status. Unsupervised clustering of protein/phosphosite signatures and pathways significantly enriched are reported on the left (*P* < 0.05; Fisher’s exact test). **b**, Association of tumor grade with functional PG subtypes. Bars indicate standardized residuals (chi-squared test). The number (*n*) of PG samples is as in **a**. **c**, Association of *BRAF* status with functional subtypes of PG-LGG (*n* = 82 PG-LGG samples). Bars indicate standardized residuals (chi-squared test). **d**, Heat map showing the 150 highest scoring proteins (top) and phosphosites (bottom) of functional subtypes in CPTAC-BRCA (two-sided MWW test). Rows are proteins/phosphosites and columns are tumors (*n* = 118 BRCA samples). Left and top tracks indicate functional subtypes; middle track indicates NMF multi-omics classification of CPTAC-BRCA (I, inclusive); and bottom track indicates tumor grade. Unsupervised clustering of protein/phosphosites signatures and pathways significantly enriched are reported on the left (*P* < 0.05; Fisher’s exact test). **e**, Association of NMF-based BRCA with functional subtypes. Circles are color coded and their size reflects the standardized residuals (chi-squared test). Orange-to-blue scale indicates positive to negative enrichment. The number (*n*) of BRCA samples is as in **d**. **f**, Heat map showing the 150 highest scoring proteins (top) and phosphosites (bottom) of functional subtypes in CPTAC-LSCC (two-sided MWW test). Rows are proteins/phosphosites and columns are tumors (*n* = 106 LSCC samples). Left and top tracks indicate functional subtypes; middle track indicates the NMF multi-omics classification of CPTAC-LSCC; bottom track indicates tumor grade. Unsupervised clustering of protein/phosphosites signature and pathways significantly enriched are reported on the left (*P* < 0.05; Fisher’s exact test). **g**, Association of NMF-based LSCC with functional subtypes. Circles are color coded and their size reflects the standardized residuals (chi-squared test). Orange-to-blue scale indicates positive to negative enrichment. The number (*n*) of LSCC samples is as in **f**. **h**, Grid plot showing top-scoring MKs common to each functional GBM, PG, BRCA and LSCC subtype (GBM, *n* = 85 samples; PG, *n* = 104 samples; BRCA, *n* = 118 samples; LSCC, *n* = 106 samples). Dots are colored according to kinase activity and their size reflect the significance of the differential activity in each group (effect size > 0.3 and *P* < 0.01; two-sided MWW test). All asterisks in **e**,**g** indicate standardized residuals higher than 1.5.

**Extended Data Fig. 7:**
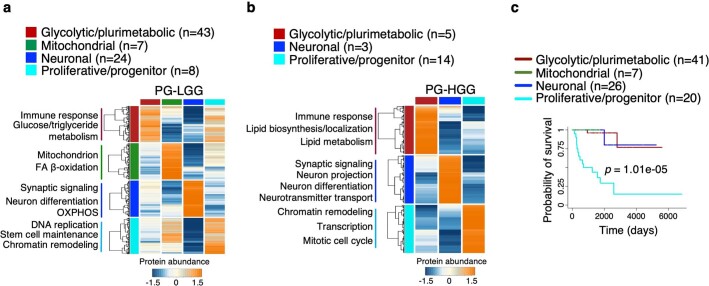
Proteomics characterization and clinical outcome of PG stratified according to functional subtypes. **a-b**, Heat map showing the median abundance of the 150 highest scoring proteins of the ranked lists (two-sided MWW test) of the four functional subtypes in a, PG-LGG and b, PG-HGG (two-sided MWW test). Rows are proteins and columns are functional subtypes (*n* = 82 PG-LGG samples; *n* = 22 PG-HGG samples). Left and top color tracks indicate functional subtypes. Unsupervised clustering was performed for each subtype-specific protein signature. For each subtype, biological pathways significantly enriched by each gene subcluster are reported on the left (*P* < 0.05, Fisher’s exact test). **c**, Kaplan–Meier curves of PG (*n* = 94 patients) stratified by SNF combining gene and protein signatures obtained from the functional GBM subtypes. Patients in the PPR subgroup exhibit significantly worse survival (log-rank test).

For PG, we integrated protein and gene expression data of 103 samples classified as high-grade (PG-HGG) or low-grade (PG-LGG) gliomas using SNF (Supplementary Tables 1d and 6a). We identified four subtypes of PG, recapitulating the functional classifier of GBM for proteomic, phospho-proteomic and gene expression data (GPM, MTC, PPR and NEU; Fig. 7a and Supplementary Table 6b–g). PG-HGG mostly clustered within the PPR subtype, whereas PG-LGG was distributed across the four subgroups (Fig. 7a,b). When PG-HGG and PG-LGG were analyzed independently for differential protein abundance, high- and low-grade tumors clustered into three and four groups, respectively, with the MTC subtype excluded from PG-HGG (Extended Data Fig. 7a,b and Supplementary Table 6h–k). BRAF *KIAA1549-BRAF* fusions and *BRAF-V600E* mutation are common in PG-LGG^49^. Glioma harboring *BRAF-V600E* were mostly classified as MTC, whereas PG-LGG harboring *KIAA1549-BRAF* fusion or *BRAF* wild-type were enriched with GPM and NEU tumors, respectively (Fig. 7a,c). Kaplan–Meier and log-rank test demonstrated significantly worse survival for the PPR subtype, a finding compatible with the predominant contribution of high-grade tumors to this group (Extended Data Fig. 7c).

**Extended Data Fig. 8:**
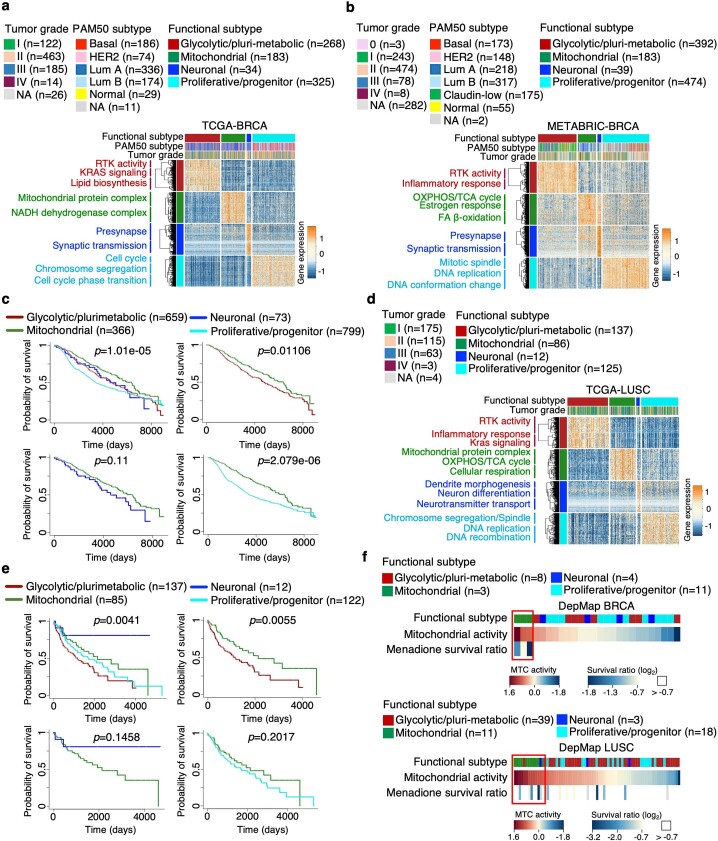
Functional classification of BRCA and LSCC and prognostic implications. **a-b**, Heat map showing the 150 highest scoring genes of the ranked lists of the four functional subtypes obtained from tumors classified in **a**, TCGA- (*n* = 810 BRCA samples) and **b**, METABRIC-BRCA (*n* = 1,088 BRCA samples) datasets (two-sided MWW test). Rows are genes and columns are tumors. Horizontal top and left tracks indicate functional subtypes; horizontal middle track indicates PAM50 classification of BRCA by TCGA; horizontal lower track indicates tumor grade. Unsupervised clustering was performed for each subtype-specific gene signature. Biological pathways significantly enriched by each gene subcluster are reported on the left (*P* < 0.05; Fisher’s exact test). **c**, Kaplan–Meier curves and log-rank test analysis of 1,897 BRCA patients from the combined TCGA (*n* = 809 patients) and METABRIC datasets (*n* = 1,088 patients), stratified according to the four functional subclasses (log-rank test). **d**, Heat map showing the 150 highest scoring genes of the ranked lists of the four functional subtypes in LUSC from TCGA database (*n* = 360 LUSC samples; two-sided MWW test). Rows are genes and columns are tumors. Horizontal top and left tracks indicate functional subtypes; horizontal lower track indicates tumor grade. Unsupervised clustering was performed for each subtype-specific gene signature. For each subtype, biological pathways significantly enriched by each gene subcluster are reported on the left (*P* < 0.05; Fisher’s exact test). **e**, Kaplan–Meier curves of 356 patients with LUSC from the TCGA dataset stratified according to the four functional subclasses. **f**, Mitochondrial activity (NES) and menadione survival ratio (log_2_) for 26 BRCA (upper plot) and 71 LUSC (lower plot) cell lines from DepMap. Upper track, functional classification; middle track, mitochondrial activity; lower track, menadione survival ratio. Survival ratio: difference between mitochondrial cell lines versus the others; log_2_(FC) = −1.31, *p* = 0.008 for BRCA; log_2_(FC) = −0.63, *p* = 0.076 for LUSC; two-sided *t*-test, unequal variance.

We also classified 118 BRCA samples into four subtypes having coherent gene expression, protein and phospho-protein abundance signatures. The three major groups represented 95% of the samples (GPM, PPR and MTC), whereas the NEU group included only five tumors (Fig. 7d and Supplementary Tables 1e and 7a–g). We found a striking association of the HER2-I (I, inclusive as defined by integrative CPTAC analysis) subgroup with the GPM subtype, Basal-I with PPR, LumA-I with MTC and LumB-I with NEU (Fig. 7e). Enrichment of HER2-I in the GPM subtype is consistent with hyperactivation of mTOR and a metabolic shift from aerobic respiration to glycolysis in this BRCA subtype^50^. The stability of the functional classification of BRCA was verified using TCGA and METABRIC gene expression data, thus authenticating the biological activities as general features for BRCA categorization (Extended Data Fig. 8a,b and Supplementary Tables 1f,g and 7h–k). The positive association between PPR and Basal-I subtype was further supported by the strong enrichment of DNA replication and proliferation-associated pathways in the Basal-I subtype (Fig. 7d). Consistent with the prolonged survival of LumA-I, the MTC-BRCA subtype had a significantly better prognosis (Extended Data Fig. 8c).

Finally, we used the functional classifier to segregate 106 LSCC tumors and tested the association with the five known LSCC-specific molecular NMF-based subtypes described by CPTAC (Fig. 7f,g and Supplementary Tables 1h and 7l–r). LSCC tumors were classified into two major subtypes (GPM and PPR) and a much smaller MTC subgroup. In this limited dataset we did not identify NEU tumors. We found a positive correlation of the MTC subtype with the Basal-I subgroup. EMT and inflamed secretory LSCC subtypes as two independent groups were functionally unified by the activation of immune, epithelial-to-mesenchymal transition (EMT) and angiogenesis functions of the GPM subtype. The PPR subtype included proliferative-primitive and classical subtypes, both sustained by proliferative-related pathways (Fig. 7f,g)^48,51^. The robustness of the functional subtyping was validated in the TCGA-LUSC (lung squamous carcinoma) dataset (Extended Data Fig. 8d and Supplementary Tables 1i and 7s,t). In this larger cohort, 12 tumors exhibited activation of synaptic functions, a hallmark of the NEU subtype. MTC-LUSC tumors exhibited more favorable clinical outcomes, suggesting that also in this tumor type OXPHOS activation produces a less aggressive biology and/or increases sensitivity to therapy (Extended Data Fig. 8e)^5^. Dependency of BRCA and LUSC MTC cells on mitochondrial activity was supported by the association between MTC activity of BRCA and LUSC cell lines in the DepMap dataset^52^ and sensitivity to menadione, a cytotoxin that specifically targets mitochondria (Extended Data Fig. 8f).

**Extended Data Fig. 9:**
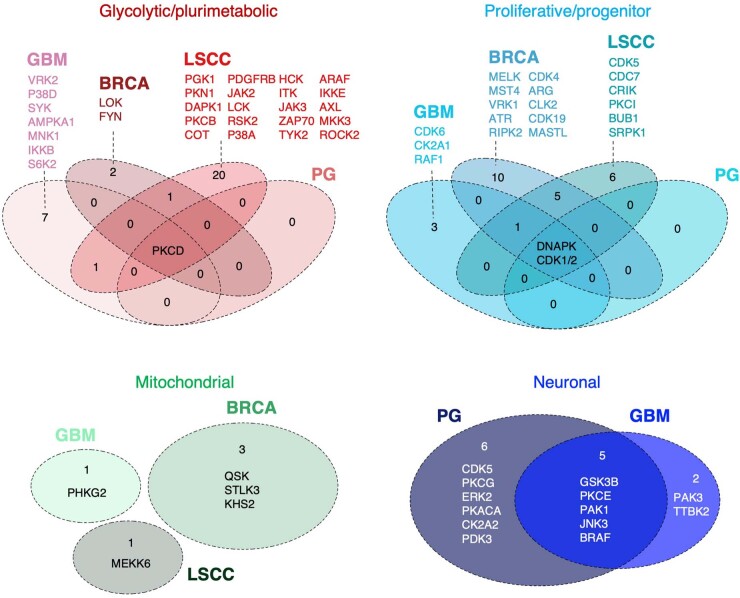
Common and specific Master Kinases across CPTAC-GBM, -PG, -BRCA, and -LSCC. Venn diagrams reporting the common and specific master kinases of each functional subtype resulted significantly activated in CPTAC-GBM, -PG, -BRCA, and -LSCC (GBM: *n* = 85 samples; PG: *n* = 104 samples; BRCA: *n* = 118 samples; LSCC: *n* = 106 samples).

Next, we applied SPHINKS to generate tumor-specific kinase–phosphosite interactomes for PG, BRCA and LSCC, including 669, 1,399 and 1,985 kinase–phosphosite relationships from 76, 198 and 103 kinases and 210, 1,899 and 699 phosphosites for PG, BRCA and LSCC, respectively and identified subtype-specific MKs (Supplementary Tables 8–10 and Extended Data Fig. 9) that we validated by global protein abundance and mRNA expression (Supplementary Tables 8–10). Most subtype-specific MKs were activated only in one tumor type (Extended Data Fig. 9). Among top-ranking tumor-specific MKs, FYN was MK of the GPM subtype in BRCA. FYN is a member of the SRC family of kinases driver of EMT in breast cancer^53,54^. VRK1 was among the top-ranking PPR MKs in BRCA. VRK1 is a chromatin-associated kinase that regulates cell cycle events and DDR previously proposed as therapeutic target in combination with DNA damage inducing therapy^55,56^. Nine protein kinases emerged as top-ranking subtype-specific MKs common to GBM, PG, BRCA and LSCC. Among them, PKCδ scored as pan-GPM and DNA-PKcs as pan-PPR MKs (Fig. 7h).

## Development of a probabilistic functional classifier of GBM

**Fig. 8:**
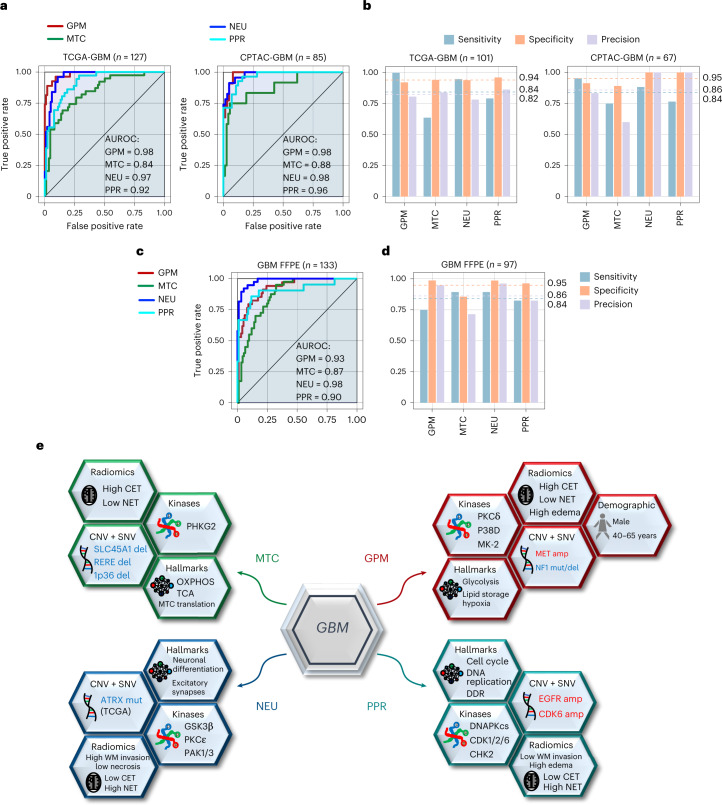
Probabilistic classifier for the identification of functional tumor subtypes of IDH wild-type GBM and schematic multi-omics and clinical modules of functional subtypes of GBM. **a**, GBM subtype-specific ROC curves for the multinomial regression model using RNA-seq data from frozen samples. Validation includes RNA-seq data from TCGA (left) or CPTAC (right) GBM samples. The number (*n*) of GBM samples for each dataset is indicated. **b**, Comparison bar plot of sensitivity, specificity and precision in each GBM subtype of the multinomial regression model as in **a**. Dashed lines and corresponding values indicate the average of each performance measure (blue, sensitivity; orange, specificity; purple, precision) in each GBM subgroup. The number (*n*) of GBM samples for each dataset is indicated. **c**, GBM subtype-specific ROC curves for the multinomial regression model using RNA-seq data from FFPE samples. Validation includes RNA-seq obtained from FFPE tumor samples. The number of GBM samples for each dataset (*n*) is indicated. **d**, Comparison bar plot of sensitivity, specificity and precision in each GBM subtype of the multinomial regression model as in **c**. Dashed lines and corresponding values indicate the average of each performance measure (blue, sensitivity; orange, specificity; purple, precision) in each GBM subgroup. The number (*n*) of GBM samples for each dataset is indicated. **e**, Functional activities, genetic alterations, MKs, clinical characteristics, radiomic features and therapeutic vulnerability compose modules that distinguish each functional subtype. GBM driver genes in each module recapitulate the functional hallmark of each subtype (for example, CDK6 amplification/CDKN2A deletion for the PPR proliferation/stemness features; MET amplification/NF1 deletion for glycolysis/RAS pathway activation in GPM GBM; FGFR3-TACC3 fusion for mitochondrial activation in MTC tumors). GPM is the only subtype that significantly associates with a specific sex (male) and age group (45–65 years). GPM and MTC subtypes exhibit positive correlation with frontal/parietal and temporal tumor location, respectively. GPM, PPR and NEU are linked with radiologic features that are compatible with the biological traits of these subgroups (CET, NET and DWM invasion, respectively). In agreement with the enhanced OXPHOS and MK activity of PKCδ and DNA-PKcs in MTC, GPM and PPR, respectively, these subtypes are distinctly sensitive to mitochondrial, PKCδ and DNA-PKcs inhibitors. CET, contrast-enhancing tumor; NET, non-contrast-enhancing tumor; DWM, deep white matter).

**Extended Data Fig. 10:**
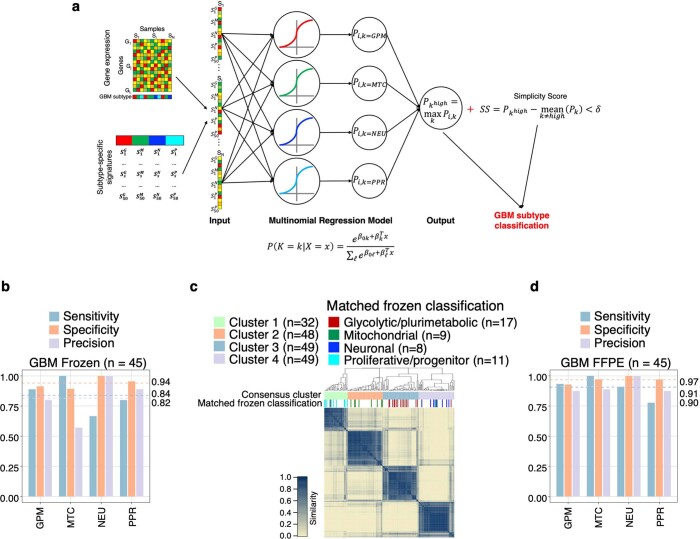
Clinical-grade probabilistic tool for the classification of frozen and FFPE IDH wild-type GBM. **a**, Schematics of the approach for calculating the probability of a GBM sample of belonging to one of the four defined functional subtypes. The Agilent expression data of 506 samples from the TCGA cohort of GBM were classified into one of the four functional subtypes (top left). The standardized expression of all the genes from the subtype-specific gene signatures (bottom left) was used to train a multinomial regression model with lasso penalty using glmnet (middle part). Each sample (input) was used to build a multi-class logistic regression model that returns four probabilities *P*_*i,k*_, one for each functional GBM subtype. We classified a tumor into one subtype if the fitted probability of the particular subtype was the highest ($P_{k^{high}}$) and the sample showed a simplicity score (SS) above a defined threshold (δ). Tumors that did not comply with the defined thresholds remained unclassified. **b**, Comparison bar plot of sensitivity, specificity, and precision in each GBM subtype of the multinomial regression model using RNA-Seq data from 45 matched frozen samples. **c**, Consensus clustering generated from the 178 FFPE GBM samples using the expression of the 200 genes from the FFPE-specific gene signatures. Columns and rows represent FFPE samples. Color bar on the top defines four subgroups according consensus clustering. Track at bottom indicates the functional classification of the corresponding 45 matched frozen samples. The number (*n*) of samples in each cluster and subtype is indicated. Yellow-to-blue scale indicates low to high similarity. **d**, Comparison bar plot of sensitivity, specificity, and precision in each GBM subtype of the multinomial regression model using RNA-Seq data from 45 matched FFPE samples. Dashed lines and corresponding values indicate the average of each performance measure (blue: sensitivity; orange: specificity; purple: precision) in each GBM subgroup.

We designed an algorithm for the probabilistic classification of individual tumors into GBM functional subtypes. When compared to RNA derived from fresh frozen samples, FFPE-extracted RNA is characterized by lower quality, typically affecting different mRNA species to variable extent^57^. Thus, we tested two classifiers, one informed by RNA-seq data from frozen tumor samples (‘frozen model’) and the other by RNA-seq data from FFPE tumors (‘FFPE model’). For the frozen model, we trained the classifier using the multinomial regression model with lasso penalty on the TCGA IDH wild-type GBM dataset profiled by Agilent expression array, which we had classified in previous work (Extended Data Fig. 10a and Supplementary Table 11a)^5^. As a feature set, we selected the 50 highest ranking genes for each functional subtype (a total of 200 gene features)^5^. To extract a reduced number of features that maximize the distinctiveness of the phenotypes, we applied a cross-validation approach and selected the model exhibiting the lowest misclassification error (17.19% cross-validation error and 6.32% error on the training set), obtaining 103 gene features with positive or negative coefficients (Supplementary Table 11b). We classified a tumor sample when the fitted probability was the highest and the simplicity score was above a predefined threshold (Methods). We tested the prediction ability of the ‘frozen classifier’ using 127 GBM from TCGA and 85 GBM from CPTAC profiled by RNA-seq. We classified 80% and 79% of the TCGA and CPTAC-GBM, respectively. The diagnostic ability of the classifier was confirmed by the AUROC of each subtype above 0.85 in each validation dataset (Fig. 8a). We determined the accuracy of the assignment of each tumor to the correct subtype^58^. Misclassification error was < 18%, sensitivity approached 85%, specificity was close to 100% and precision > 80%, indicating a robust performance of the classifier (Fig. 8b and Supplementary Table 11c). The frozen model was validated on an independent cohort of 45 frozen samples for which matched FFPE samples were available (see below), obtaining similar results (Extended Data Fig. 10b).

For the FFPE model, to account for the lower quality of FFPE-extracted RNA, we sequenced the transcriptome of 45 frozen and FFPE matched samples and selected 4,668 genes that exhibited consistent expression profiles in both sample types (genes supposedly unaffected by FFPE treatment, Spearman correlation, *ρ* > 22; Supplementary Table 12). With the classification of frozen samples as the gold standard, we generated subtype-specific signatures using expression profiles of the corresponding FFPE samples. We then trained the multinomial regression model using FFPE-specific signature genes from TCGA-GBM Agilent cohort (66 gene features, 19.76% cross-validation error and 11.07% error on the training set). The performance of the classifier was assessed on an independent cohort of 133 FFPE samples profiled by RNA-seq, classifying 73% of the samples. To assess the stability and accuracy of the FFPE model, we unbiasedly assigned FFPE samples to a subtype by unsupervised consensus clustering of 178 samples (133 FFPE plus 45 FFPE with matched frozen specimens; Extended Data Fig. 10c). Using the classification of the 45 frozen samples as ‘anchors’, we assigned each cluster to a functional GBM subtype and compared the resulting unbiased label assignment with the subtype classification from the FFPE model for the 133 unmatched FFPE samples only. The classifier performance indexes were similar to those calculated for the frozen model (misclassification error of 15%; AUROCs, sensitivity, specificity and precision > 0.84; Fig. 8c,d and Supplementary Table 11b,c). The FFPE model was also validated on 45 FFPE samples using the classification of the matched frozen specimens as ground truth, obtaining comparable results (Extended Data Fig. 10d).

We have implemented a Shiny app of the frozen and FFPE classification tools for general research use at https://lucgar88.shinyapps.io/GBMclassifier.

## Discussion

Here, we sought to establish a link between multi-omic features that regulate the biology of GBM subtypes and protein kinases that could directly enable subtype-specific phenotypes. We built and applied SPHINKS-MK, an algorithm that integrates proteomics and phospho-proteomics datasets into a single network for the unbiased extraction of subtype-specific MKs. By informing pharmacologic and genetic experiments in subtype-matched GBM organoids, SPHINKS-MK delivered PKCδ and DNA-PKcs as experimentally validated MKs for the aggressive GPM and PPR subtypes of GBM. The four subtypes and the underlying phenotypes were also recovered across different tumor types, highlighting the fundamental biological traits that are extracted by the functional classification. In the multi-cancer context, PKCδ and DNA-PKcs have emerged as broadly actionable MKs of GPM and PPR subtypes. Inspired by the subtype-specific therapeutic opportunities, we present a probabilistic classifier that enables rapid translation of precision therapeutics for subgroups of patients with GBM.

The four GBM subtypes initially inferred from a pathway-based scRNA-seq analysis are supported by orthogonal analyses from proteomics, phospho-proteomics, metabolomics, lipidomics and acetylomics platforms. The divergent metabolism of the GPM and MTC subtypes was independently captured by the analysis of acetylomics, a post-translational modification previously associated with the inactivation of metabolic proteins^25^. Acetylation also emerged as major determinant factor instructing the identity of the proliferation-, stemness- and DDR-related biology that is activated in PPR cells. Stratification of PPR GBM based on acetylation of nuclear proteins uncovered a hyperacetylated PPR group of tumors with outlier activation of these activities. This finding underscores the crucial role of acetylation of nuclear proteins for activation of transcription and chromatin-remodeling factors and enzymes involved in the DDR^59^. The significance of the pathway-based classification of GBM is further emphasized by the association of the individual subtypes with clinical variables such as age and tumor location within the central nervous system and frequency of recurrent alterations of driver genes. The interrogation of MRI features associated with each subtype showed that the metabolic subtypes, and particularly the GPM subgroup, are characterized by higher contrast enhancement, potentially reflecting more prominent perivascular invasion of tumor cells with consequent disruption of the endothelial tight junctions of the blood–brain barrier. Conversely, tumors classified along the neurodevelopmental axis are associated with non-enhancing features. Among them, the unique correlation between NEU tumors and deep white matter invasion is consistent with the proposed ability of neuronally differentiated GBM cells to engage healthy brain cells at the tumor periphery for neomorphic synaptic connections that guide invasion through white matter tracks^5^ (Fig. 8e).

Although prediction of active protein kinases in cancer has been so far of limited impact for cancer therapy, there is tremendous appeal of kinases as both drivers and drug targets. SPHINKS-MK interrogated the full scope of tumor-specific kinomes and phosphorylomes reconstructed into an integrated functional network and identifies high-activity kinases specific for tumor subtypes. The benchmarking of SPHINKS showed that the algorithm is stable and exhibits a prediction power higher than other inference methods. PKCδ emerged as the top-scoring kinase of the GPM subtype. Genetic and pharmacologic inhibition of PKCδ defined its role in oncometabolic processes at the intersection of insulin, IGF and lipid metabolism and validated PKCδ as crucial therapeutic target of the GPM subtype of GBM. DNA-PKcs was experimentally validated as essential MK of the PPR subtype. The synergistic and lethal effect of inhibition of DNA-PKcs and IR in PPR but not GPM cells provided the mechanistic interpretation of therapy resistance in this GBM subtype. As DNA-PKcs inhibitors have been introduced into clinical trials^44,60^, our findings indicate that preselection of patients with PPR tumors is likely to enhance therapeutic success. The GBM classifier was validated as a stratifying method for pediatric and adult tumors, revealing consistent patterns across different tumor types (for example, favorable survival associated with MTC tumors) and context-dependent features (BRAF mutations and fusions associated with divergent metabolic subtypes in PG). The identification of PKCδ and DNA-PKcs as subtype-specific MKs from SPHINKS-inferred PG, BRCA and LSCC kinase–phosphosite interactomes delivers targeted therapeutic directions for GPM and PPR subtypes across multiple tumor types.

The probabilistic classification tool will facilitate the yet unfulfilled stratification of patients with GBM for the accrual to clinical trials using FFPE specimens and advance precision therapies targeting individual subtypes of this aggressive tumor.

## Methods

### Ethics statement

PDOs have been described previously^5^. PDOs were obtained using excess material collected for clinical purposes from specimens de-identified at the source. This work was designated Institutional Review Board exempt under paragraph 4 and covered under Institutional Review Board and Onconeurotek tumor bank certification (NF S96 900) and authorization from an ethics committee (CPP Ile de France VI, ref. A39II) and the French Ministry for Research (AC 2013–1962).

### Patient datasets and profiling platforms

For each cancer type^6,9,46–48,61–63^, multi-omics data availability, tumors analyzed, clinical and survival data are listed in Supplementary Table 1.

### Data processing

#### Gene expression

Data from CPTAC were downloaded as fpkm. Non-protein-coding and low-expressed genes were removed. Data were quantile and log_2_ normalized. Data from METABRIC (Illumina HT-12 v.3) were downloaded as median normalized. RNA-seq data from TCGA were downloaded using TCGAbiolinks. Upper quantile within-normalization with GC content correction and between-normalization were applied.

#### DNA methylation

Data from CPTAC (EPIC array) were downloaded as β-values, pre-processed with functional normalization with minfi^64^, quality checked, with common single-nucleotide polymorphism filtering and probe annotation. Values missing in < 20% across all sample were imputed using the average of the corresponding probe. Data from TCGA were pre-processed with functional normalization and probes targeting sex chromosomes or not associated with gene promoters^65^ were removed. Processed β-values and classification of the MNP cohort were downloaded from the Gene Expression Omnibus (GSE90496, MNP reference set) and supplementary tables published previously^9^.

#### Copy number

Thresholded CNVs were assessed using GISTIC. Protein-coding genes were retained. *f*CNVs were obtained as described^5^.

#### Global proteome and phospho-proteome

Values missing in <50% across all samples were imputed with DreamAI^66^ and were quantile and log_2_ normalized.

#### Lipidome and metabolome

Data were downloaded as log_2_-tranformed and median normalized. Values missing in fewer than five or ten tumors for lipids or metabolites, respectively, were imputed using average abundance of the corresponding molecule. Data were quantile normalized.

#### Acetylome

Data were imputed with DreamAI and log_2_-transformed.

### Functional classification of CPTAC IDH wild-type GBM

We used Agilent expression profiles of 304 TCGA-GBM IDH wild-type previously classified^5^ as training set of a *k*-nearest neighbors (*k*-NN) classifier (*k* = 3) to classify CPTAC tumors. To account for differences in gene expression between TCGA and CPTAC, we generated ranked lists of genes differentially expressed in each CPTAC subtype compared to the others using the MWW test and defined as subtype-specific signatures the 50 highest scoring genes. For each tumor, we derived the intensity of each subtype as the average expression of genes in each subtype-specific signature. A simplicity score was obtained as the difference between the two highest subtypes intensities, and tumors with simplicity score > 0.6 were retained (17 GPM, 6 MTC, 16 NEU and 13 PPR core samples).

To assign membership to 40 unclassified tumors, we integrated *f*CNV and gene expression using SNF for 89 tumors. The features set of the classifier (subtype-specific *f*CNV gains/losses from TCGA and subtype-specific gene signatures from CPTAC core samples) were aggregated by SNFtool to generate a fused tumor network and a tumor similarity matrix (*K* = 20, *α* = 0.5 and *t* = 20). Spectral clustering was performed on the similarity matrix. The distance matrix (1 − similarity) was used to establish membership of 38 unclassified GBM according to the closeness to core tumors with *k*-NN (*k* = 3). Five tumors with conditional probability < 0.6 remained unclassified.

### Cross-classification analysis

We classified TCGA- and CPTAC-GBM samples according to MNP DNA methylation classification^9^ using MNP-GBM and assignment as training set of *k*-NN. The top 10,000 variable probes shared by MNP and TCGA or CPTAC samples were selected. We extracted the top 30 principal components by principal-component analysis and assigned an MNP classification to TCGA or CPTAC samples using *k*-NN (*k* = 9)^6^. While an official MNP classifier exists online (https://www.molecularneuropathology.org/mnp), we were not able to access it as the site did not approve our registration at the time of writing.

To assess the relationship between pathway-based classification and transcriptional subtyping in TCGA- and CPTAC-GBM, we analyzed 304 TCGA-GBM previously classified^5^. TCGA subtype assignments were obtained as described^8^. Subtyping of CPTAC tumors was described previously^6^.

### Multi-omics characterization of GBM functional subtypes

We generated ranked lists of genes, proteins, lipids and metabolites differentially expressed/abundant in each subtype compared to the others by MWW test. Final subtype-specific signatures including the 150 top-scoring genes or proteins were used to calculate subtype enrichment in each tumor using single-sample MWW-GST (ssMWW-GST). Pathway enrichment analysis was performed as described elsewhere^5^, using non-redundant pathways from a set cover algorithm^67^. The most active pathways in each subtype were obtained using gene or protein ranked lists by two-sided MWW-GST (logit(NES) > 0.58, FDR < 0.005).

Enrichment of glycolytic and mitochondrial enzymes (protein sets) and metabolic intermediates (metabolite sets) in MTC and GPM were generated by MWW-GST (glycolytic enzymes: logit(NES) = 1.27, *P* = 0.017; mitochondrial enzymes: logit(NES) = −1.19, *P* = 5.93 × 10^−13^; glycolytic intermediates: logit(NES) = 1.76, *P* = 0.0007; mitochondrial intermediates: logit(NES) = −1.65, *P* = 0.018). The network of metabolites and metabolic proteins was constructed using Ingenuity Pathway Analysis (IPA)^68^.

Lipid signatures included molecules with an MWW score > 0.5. Lipids were categorized and used for enrichment of lipid subclasses, cellular components and lipid functions in each subtype using Fisher’s exact test (FET; log(OR) > 0, *P* < 0.05) and the lipid ontology database LION^14^.

### Proteogenomic integrative analysis of GBM

*f*CNV^prot^ were obtained by integrating *f*CNVs, gene expression, and protein abundance of genes that exhibited *f*CNV change in two or more tumors according to the following criteria: (1) higher/lower protein abundance in tumors with alteration compared to wild-type (|log_2_ (*FC*)| > 0.15, *P* < 0.10; two-sided MWW test); (2) higher/lower protein abundance in one subtype compared to the others (|log_2_ (*FC*)| > 0.15, *P* < 0.10; two-sided MWW test); (3) higher subtype-specific transcriptomic activity of tumors harboring the *f*CNV compared to wild-type (effect size > 0.15, *P* < 0.10; two-sided MWW test). Subtype-associated *f*CNV^prot^ gains/losses were examined for their contribution to activation/deactivation of biological pathways using FET (*P* < 0.05).

#### Univariate logistic regression analysis

Tumors were segregated according to *f*CNV status (altered, wild-type); subtype activity was a continuous predictor. Additionally, tumors were segregated according to subtypes and protein abundance was used as a continuous predictor. The analysis of FGFR3-TACC3 fusion included 178 GBM FFPE RNA-seq samples (fusion present, 12 tumors or absent).

### Analysis of acetylation of metabolic and nuclear proteins

We used 2,212 genes from the Reactome Metabolism gene set to define proteins involved in metabolism. Unsupervised clustering was performed on proteins differentially expressed between GPM and MTC (*P* < 0.05, log_2_(FC) > 0.3; two-sided MWW test).

Normalized acetyl site abundance (acetylation not explained by the corresponding protein abundance) was calculated as residuals (*ε*_site_) from the linear regression *Ac*_site_ = *β*_0_ + *β*_1_ × Pr_site_ + *ε*_site_, where *Ac*_site_ is the abundance of a given acetyl site and *Pr*_site_ is the corresponding protein abundance.

We applied BlackSheep’s differential extreme value analysis module to define outlier acetylated metabolic proteins (*P* < 0.05) and enrichment of biological pathways using FET (*P* < 0.0005).

Nuclear proteins were selected by the COMPARTMENTS database^69^ (nucleus score of 5). Acetyl sites with the highest variability across the dataset by interquartile range (*n* = 320) were used for unsupervised clustering. Differentially abundant acetyl sites in high- versus low-acetylation PPR subgroups were defined by MWW test (*P* < 0.001, log_2_(FC) > 0.3). Acetyl sites whose abundance was not explained by protein abundance were selected by comparing global protein and acetyl site abundance between high- and low-acetylation PPR subgroups using MWW test (log_2_(FC) > 0.5, *P* < 0.05). Pathway overrepresentation testing was performed using gProfiler tool (FDR < 0.05).

### Generation of replication stress/DNA damage response phospho-proteomic signature

We manually curated data from five studies reporting mass spectrometry phospho-proteomics^70–74^ to identify sites whose phosphorylation was increased after induction of DNA RS by ATR inhibition or hydroxyurea treatment or DDR by IR exposure. Differential abundance of DDR/RS-induced-phosphosites was performed comparing PPR subgroup versus the others (*P* < 0.05; MWW test). DDR/RS phospho-signatures were used to calculate DDR/RS scores in each tumor (ssMWW-GST). Enrichment of GPM, MTC, NEU and PPR tumors in highest/lowest distribution of the DDR/RS score (|logit(NES)| > 0) was tested using FET. Difference between transcriptome- and global proteome-derived subtype activity was calculated and the association with DDR/RS score tested using Spearman’s correlation.

### Functional classification, analysis and validation of PG, BRCA and LSCC

We used RNA-seq expression profiles of 105 CPTAC-PG, 119 CPTAC-BRCA and 108 CPTAC-LSCC to compute the enrichment of the functional subtype-specific signatures from TCGA-GBM in each tumor and protein abundance data to compute the enrichment of the 50 highest scoring proteins in the ranked list of each CPTAC-GBM subtype in each tumor using ssMWW-GST. Tumors were classified according to the subtype with the concordant highest NES (logit(NES) > 0.3, FDR < 0.05) in both transcriptomic and proteomic data and were defined as ‘anchor tumors’ (51, 54, 64 tumors for PG, BRCA and LSCC, respectively). We used anchor tumors to generate ranked lists of genes and proteins (MWW test). Tumor type-specific/subtype-specific gene and protein signatures included the top 50 scoring genes and proteins. Unclassified tumors (54 PG, 96 BRCA and 44 LSCC) were classified by integrating gene and protein signatures from the previous step using SNF. Final classifications include 48 GPM, 7 MTC, 27 NEU, 22 PPR and 1 unclassified for PG; 50 GPM, 23 MTC, 5 NEU and 40 PPR for BRCA; and 51 GPM, 9 MTC, 0 NEU, 46 PPR and 2 unclassified for LSCC samples. We used the expression profiles of 1,095 tumors from TCGA-BRCA, 1,904 tumors from METABRIC-BRCA and 502 tumors from TCGA-LUSC to compute the enrichment of functional subtype-specific signatures from TCGA-GBM in each tumor (ssMWW-GST), classifying them according to the subtype with the highest NES (logit(NES) > 0.58, FDR < 0.05).

Normalized phosphosite abundance (phosphorylation not explained by the corresponding protein abundance) was calculated as for normalized acetyl site abundance, using the abundance of the phosphosite and corresponding protein.

Association between functional classification and tumor grade, BRAF status (PG) or CPTAC NMF-derived subtypes (BRCA and LSCC) was assessed by chi-squared test. Survival analysis among functional subtypes in TCGA-BRCA, TCGA-LUSC and METABRIC-BRCA was assessed by log-rank test.

### DepMap data analysis

Transcriptomic profiles of BRCA and LUSC cell lines from DepMap for which both RNA-seq expression and menadione survival ratio from PRISM Repurposing Primary Screen were available (BRCA, *n* = 26; LUSC, *n* = 71)^52^ were used to derive subtype activities and classification according to the highest NES (ssMWW-GST). Difference in menadione survival ratio between MTC cell lines versus the others was assessed using two-sided *t*-test, unequal variance.

### SPHINKS algorithm

We implemented SPHINKS, a machine-learning method that generalizes unseen data from observed data using semi-supervised approaches applied in gene regulatory networks reconstruction^75^. SPHINKS creates an unbiased context-specific kinome network, leveraging kinases abundance from proteomics, substrate abundance from phospho-proteomics and experimentally validated kinase–substrate interactions available from PhosphoSitePlus^30^. The classifier, as a binary model, was trained to recognize relationships between abundance profiles of kinase–phosphosite pairs. A positive training set was defined as the set of known substrates of a specific kinase. This represented the typical setting where a learner has access only to positive and unlabeled data (positive unlabeled)^75^, with high imbalance between positive and unlabeled examples. We combined easyensamble^76^ and bootstrap aggregating machine-learning ensemble meta-algorithm (bagging)^77^ to integrate several SVM classifiers trained on different instances of the negative set (Extended Data Fig. 4a). An SVM classifier was trained on the validated interactions (positive training set) and a subset of randomly selected unknown interactions (negative set). Each training example represents an interaction and a training matrix is formed juxtaposing kinase’s protein and substrate’s phospho-protein abundance on a set of corresponding cases, with examples along the rows. Using the matrix of all possible kinase–substrate pairs we obtained a score (between 0 and 1), representing the probability for each phosphosite to be a kinase substrate according to the classifier. As the randomly derived negative set may contain potential substrates, to improve the accuracy of the prediction, we applied the bagging, repeating the training/prediction steps 100 times using random sampling of the negative set (keeping the positive fixed). SPHINKS scores were derived as the average SVM score from all iterations. To create a set of predicted substrates (SOPS) for each kinase (a list of predicted kinase–substrate interactions), we selected interactions whose SPHINKS score was above the value for which at least 50% of the known interactions were retained and the Spearman’s correlation between kinase and phospho-substrate was positive.

### Identification of subtype-specific master kinases

We applied the method described previously^10^ with modifications. The activity of an MK was defined as the quantification of the activation of its substrate program in each sample *X*_*i*_ (i = 1,…,85). We binned all substrates into 25 bins according to their average abundance across all samples. For each MK, we defined {*s*_1_,…,*s*_*K*_} the substrates in the SOPS of MK. We randomly extracted a set of *n* = 100 control substrates for each *s*_*k*_ from the corresponding bin, {*c*_1_,…,*c*_100*K*_}. Thus, the control substrate set has a distribution of abundance levels comparable to that of SOPS, while being 100-fold larger. The activity of the MK in the sample *X*_*i*_ was computed as:

$${\mathrm{Act}}\left( {X_i,{\mathrm{MK}}} \right) = \frac{{\mathop {\sum}\nolimits_{k = 1}^K {\omega _{s_k} \times t_{s_k}^i} }}{{\mathop {\sum}\nolimits_{k = 1}^K {\omega _{s_k}} }} - \frac{{\mathop {\sum}\nolimits_{j = 1}^{100K} {\omega _{c_j} \times t_{c_j}^i} }}{{\mathop {\sum}\nolimits_{j = 1}^{100K} {\omega _{c_j}} }},$$

where *ω*_*sk*_ and *ω*_*cj*_ are the SPHINKS scores of the *k*th substrate or *j*th control substrate of the MK, respectively; $t_{s_k}^i$ and $t_{c_j}^i$ are the abundances of *s*_*k*_ or *c*_*j*_ in the *i*th sample, respectively. If Act (*X*_*i*_, MK) > 0, the MK is activated in the *i*th sample, if Act (*X*_*i*_, MK) < 0, the MK is inversely activated and if Act (*X*_*i*_, MK) ≈ 0, it is deactivated.

We selected MKs that showed a significant difference in activity in one subtype compared to the others using MWW test (effect size > 0.3 and *P* < 0.01). For GBM, subtype-specific MKs were mapped on a kinome tree using KinMap^78^.

### Benchmarking of SPHINKS

#### Impact of missing values and imputation algorithm

To establish how the SPHINKS prediction of kinase–phospho-substrate interactions degrades as the level of imputation increases, we performed a set of simulations in a controlled setting where we could have a gold standard. From the CPTAC-GBM un-imputed phospho-proteomic data, we selected sites with no missing values (*n* = 7,302) as input for SPHINKS and generated a kinase–phosphosite interactome to be used as a gold standard. To simulate missing values, we generated new phospho-proteomic datasets by randomly replacing predefined ratios of phosphosites with missing values (*r* = 10%, 25% and 50%) and then imputed using DreamAI. We applied SPHINKS to predict the networks on the imputed matrices and compared them with the one reconstructed from the un-imputed matrix. The AUC from the ROC curve was computed as a measure of accuracy.

#### Validation of the predictions of kinase–phospho-substrate interaction

To evaluate SPHINKS performance in the prediction of kinase–substrate interactions, we performed a tenfold cross-validation analysis by randomly dividing the validated interactions from PhosphoSitePlus into ten subsets for training and testing. The workflow for each fold is as follows:

1. We trained the SVM using the training subsets (positive training set) plus a random selection of unknown interactions (negative training set).
2. As test set, we used the test subset and a randomly selection of unknown interactions, completely independent from the negative training set and derived the scores using the SVM classifier from step 1.
3. We derived the SPHINKS scores by applying the bagging approach as described before, repeating step 1 and 2 100 times.
4. We compared the SPHINKS scores with the test set and derived the AUC.

#### Validation of the kinase activity estimate

To evaluate how much different levels of interaction misclassifications affect the SPHINKS kinase activity, we randomly perturbed the SPHINKS network, as follows:

1. From the predicted kinase–substrate interactome, we generated a set of perturbations of interactions by replacing a predetermined percentage of phospho-substrates corresponding to *P(percentage)* = bottom 5%, 10%, 15%, 20% and 50% of the SPHINKS scores with random phosphosites.
2. For each percentage, we randomly generated *n* = 100 runs of perturbed networks.
3. For each percentage and run, we derived the SPHINKS kinase activity for 154 kinases in 85 CPTAC-GBM samples.
4. For each percentage and run, we derived the MK Δ-activity as the difference (in percentage) between the kinase activity inferred using the original network (Act(MK)^*u*^) and the perturbed networks (Act(MK)^*P*^):

$${\Delta}Act\left( {MK} \right) = abs\left( {\frac{{Act\left( {MK} \right)^u - Act\left( {MK} \right)^p}}{{Act\left( {MK} \right)^u}}} \right).$$

Average ${\Delta}Act\left( {MK} \right)$ for each kinase across all runs or for each run across all kinases were shown at each ratio of perturbation.

#### Comparison of the kinase activity inferred by SPHINKS and other methods

We considered two recently reported approaches, KSEA^31^ and KEA3 (ref. ^32^).

We used a dataset reporting the downstream changes in phospho-protein abundance after perturbations of upstream kinase by stimulators or inhibitors^33,34^, bringing together 24 studies encompassing 103 kinase-perturbation annotations (gold standard) for 30 kinases and 61,181 phosphosites. We employed a metric defined as ‘top-k-hit’ *(P*_hit_(*k*)), which focuses on the top *k* kinase predictions, as described^34^, with *k* = 10. To compare the kinase activity estimate among methods, for SPHINKS we considered only the validated interactions.

Additionally, we evaluated whether other approaches could identify the GBM subtype-specific kinases uncovered by SPHINKS. We applied each method on the CPTAC-GBM dataset and for each subtype derived the ranking of 129 kinases included in all five methods: (1) for SPHINKS, kinases were ranked according to the MWW score; (2) for KSEA PhosphoSitePlus and KSEA PhosphoSitePlus + NetworKIN, kinases were ranked based on the KSEA-derived *z* score^31^ for each subtype compared to the others; and (3) for KEA3, kinases were ranked based on the MeanRank or TopRank^32^ for each subtype (considering the highest 300 differentially phosphorylated proteins). For each kinase, we derived the Δ-rank as the difference in ranks between SPHINKS and any other approach (Δ-rank < 0, the rank of SPHINKS is lower, indicating higher kinase activity; Δ-rank > 0 indicates the opposite).

### Processing and library preparation of the in-house GBM IDH wild-type cohort

The cohort is composed of 178 FFPE IDH wild-type GBM samples, 45 of which had matched frozen specimens. RNA was extracted using the Maxwell Rapid Sample Concentrator Instrument (Promega) and Maxwell RSC simplyRNA Tissue Kit (Promega, AS1340) for frozen samples or Maxwell RSC RNA FFPE kit (Promega, AS1440) for FFPE specimens. RNA extracted from both tissues was analyzed using the same workflow. Complementary DNA libraries were prepared with QuantSeq 3′ mRNA-Seq Library Prep kit FWD (Lexogen, 015). In brief, libraries were prepared with oligo-dT priming, with no previous poly(A) enrichment or ribosomal RNA depletion required. After first-strand synthesis, second-strand synthesis was initiated by random priming and Illumina-specific linker sequences were introduced. The resulting double-stranded cDNA was purified with magnetic beads and the library was then amplified, introducing the sequences required for cluster generation. Illumina libraries were multiplexed compatibly with single-end sequencing and sequenced on the Illumina HiSeq platform (100-bp single end). Sequencing data quality and pre-processing was as described^5^.

### Development of the probabilistic classification tool for IDH wild-type GBM

We used 506 tumors from the TCGA-GBM profiled by Agilent as training set as these tumors were assigned to each functional subtypes based on orthogonal validation across multiple platforms including *f*CNVs, somatic mutations, DNA methylation and miRNA gene signatures^5^. The standardized expression of all genes from the subtype-specific signatures was used to train a multinomial regression model with lasso penalty using glmnet (α = 1, family = ‘multinomial’)^79^. We applied a tenfold cross-validation to select the best model with the lowest cross-validation error based on the misclassification error as loss measure. As a test set (ground truth), we considered two GBM IDH wild-type RNA-seq datasets:

- TCGA-GBM cohort (*n* = 127) classified according to the subtyping of the matched Agilent expression tumors (ground truth);
- CPTAC-GBM cohort (*n* = 85) classified in functional subtypes (ground truth) as described in this manuscript and orthogonally validated by multi-omics analyses (global proteomics, phospho-proteomics, lipidomics, metabolomics and acetylomics).

We classified the test samples if the fitted probability of a particular subtype was the highest and the sample showed a simplicity score above 0.35. The simplicity score was computed as the difference between the highest fitted probability (dominant subtype) and the mean of the other subtypes (non-dominant). We classified 80% of the TCGA and 79% of the CPTAC cohorts.

For the FFPE model, we used a similar approach with some modifications. We generated RNA-seq data from FFPE of 178 IDH wild-type GBM, 45 of which were also independently sequenced from matched frozen specimens (Supplementary Table 12). To identify genes whose expression in FFPE is consistent with the corresponding frozen specimens, we calculated correlation of expression between the 45 matched frozen and FFPE samples and retained only genes with Spearman’s correlation > 0.22 (4,668 genes). Independently, we classified the 45 fresh frozen samples’ extracted RNA to each subtype on the basis of the highest NES (ssMWW-GST) using the functional subtypes signatures^5^. Using the classification of the frozen samples as a ‘gold standard’, we derived FFPE-specific subtype-specific signatures on the FFPE expression matrix (50 highest genes from each ranked list, MWW test). As described for the frozen model, we trained a multinomial regression model on TCGA Agilent cohort using the FFPE-specific gene signatures and applied cross-validation to select the best model. The remaining 133 samples that lacked RNA-seq data from frozen specimens and had not been used to define the FFPE-specific signatures were classified if the fitted probability of a particular subtype was the highest and the simplicity score was above 0.25. We classified 73% of these tumors.

We performed an independent analysis to obtain an unbiased subtype assignment of the FFPE samples. FFPE-specific gene signatures were used to inform consensus clustering on the Euclidean distance matrix of all 178 FFPE-derived RNA-seq (10,000 random samplings, 70% of samples, Ward linkage, *k* = 4 clusters). We then labeled all samples by assigning each individual cluster to each subtype using the classification of the 45 matched frozen samples as ‘anchors’. We found 91% concordance in the classification of the matched frozen and FFPE-derived RNA-seq (41 out of 45). Finally, the unbiased label assignments of 133 unmatched FFPE samples were used to evaluate the prediction abilities of the classifier.

### Association of GBM functional subtypes with clinical and radiomic features

Clinical data for TCGA-GBM patients were downloaded using TCGAbiolinks. Demographic characteristics were available for 503 GBM classified according to pathway-based classification. Patients were segregated in three age groups: 10–40, 40–65 and > 65 years. Quantification of radiomic features were available for 88 preoperative multimodal MRIs of TCGA-GBM from TCIA. For tumor location, patients were segregated in high or low group if more/less than 50% of the tumor was detected in the specific location, respectively. Univariate logistic regression analysis was performed to assess the association between demographic or radiomic features and functional subtypes/axis. Radiologist-made assessments (proportion of necrosis and edema) from TCGA (*n* = 63 GBM with available pathway-based assignment) were retrieved from elsewhere^12^. The proportion of DWM invasion available through TCIA was obtained by the integration of data published previously^13^ and REMBRANDT (*n* = 54). Quantitative radiomic features (*n* = 175) from 88 GBM were selected from TCIA as described^80^. We performed differential analysis of radiomic data in each subtype compared to the others (FC > 0.3, *P* < 0.05; two-sided MWW test). Association between functional subtypes and radiomic subgroups from unsupervised clustering was assessed by chi-squared test.

### Cell culture

PDOs were cultured and tested as described^5^. Human cell lines were HEK293T (ATCC CRL-11268). Cells were cultured in DMEM supplemented with 10% FBS (Sigma). Cells were transfected using Lipofectamine 2000 (Invitrogen) or the calcium phosphate method. Lentiviral infection was performed as described^10^. Short hairpin RNA (shRNA) sequences (Sigma) for PKCδ are:

PRKCD shRNA 1 (TRCN0000010193): GGCCGCTTTGAACTCTACCGT;

PRKCD shRNA 2 (TRCN0000379731): CATTACTTGAATGTAGTTATC;

#### Cell growth and clonogenic assay

Time course analysis of the cellular growth of sh*PRKCD* or empty vector-transduced PDOs was performed by plating 4,500 cells per well in 96-well plates. Viability was determined using CellTiterGlo assay reagent (Promega, G7570) and the GloMax-Multi+ Microplate Multimode Reader (Promega). For clonogenic assay of PDOs treated with BJE6-106, 1,500 cells were plated in six-well plates. Cells were fixed in methanol and stained with crystal violet after 2 weeks. Colonies with more than 50 cells were scored. Data are mean ± s.d. (*n* = 3 biological replicates). Experiments were repeated twice.

#### Intracellular glucose uptake and triacylglyceride accumulation

Measurement of the rate of glucose uptake and triacylglyceride accumulation in sh*PRKCD* and control infected GPM PDO cells were performed as described elsewhere^5^.

#### Compound treatment

Cells were plated in 130 μl in opaque white 96-well plates. At 24 h later, cells were treated with serial dilutions of compounds as indicated for 72 h. Viability was determined as described^5^. For IR–drug combination treatment, PDOs were plated in 96-well plates. Cells were treated 24 h later with serial dilutions of M3814 and exposed 2 h later to IR (2, 4, 8 Gy at 0.7 Gy min^−1^) from a ^137^Cs source (GammaCell 40 irradiator, Teratronics). Mock-treated cells were cultured in parallel. Viability was determined 96 h later as described above. Clonogenic assays for the evaluation of IR–drug combination were performed in three independent 96-well plates for treatment group. The number of wells containing PDO spheres was scored and normalized to untreated cells.

### Immunofluorescence analysis of γH2AX foci

Cells were fixed with 4% paraformaldehyde, permeabilized with cold methanol for 90 s at 4 °C and blocked with 5% BSA, 0.05% Triton X-100 in PBS for 30 min. Cells were exposed to primary antibody phospho-H2AX 1:500 dilution (Ser139, CST, 2577) for 1 h at room temperature followed by Cy3-conjugated anti-rabbit (Invitrogen, A10520) for 1 h at room temperature. Nuclei were stained with 4,6-diamidino-2-phenylindole (DAPI) (Sigma). Images were acquired using a Nikon Ti Eclipse inverted microscope for spinning-disk confocal microscopy equipped with a Plan Apochromat ×60 oil/1.4 NA DIC objective. **γ**H2AX foci in individual nuclei were scored by ImageJ (NIH) with in-built find Maxima > Prominence > Point Selection plug-in. Nuclei from at least ten random images were included in the analysis of each treatment group.

### Western blot

Cells were lysed in RIPA buffer (50 mM Tris-HCl, pH 7.5, 150 mM NaCl, 1 mM EDTA, 1% NP40, 0.5% sodium dexoycholate, 0.1% sodium dodecyl sulfate, 1.5 mM Na_3_VO_4_, 50 mM sodium fluoride, 10 mM sodium pyrophosphate, 10 mM β-glycerol phosphate and EDTA-free protease inhibitor cocktail; Roche). Lysates were briefly sonicated, cleared by centrifugation, separated by SDS–PAGE and transferred to polyvinylidene difluoride membrane. Membranes were probed with primary antibodies overnight at 4 °C: p-DNA-PK (Ser-2056, CST, 68716), DNA-PK (CST, 38168), p-NBS1 (Ser-343, CST, 3001), NBS1 (CST, 14956), p-KAP1 (Ser-824, Abcam, ab133440), KAP1 (Abcam, ab109287), p-CHK1 (Ser317, CST, 12302), CHK1 (CST, 2360), p-PKCδ (Tyr-311, CST, 2055), PKCδ (Abcam, ab182126), PKCδ (CST, 9616), p-STAT3 (Tyr705, CST, 9145), STAT3 (CST, 4904), p-AKT (Ser-473, CST, 4060), p-AKT (Thr308, CST, 13038), AKT (CST, 4691), p-ERK1/2 (Thr202/Tyr204, CST, 4370), ERK1/2 (CST, 9102), GAPDH (Abcam, ab9484), Vinculin (Sigma, V9131) and β-actin (Sigma, A5441). Secondary horseradish peroxidase-conjugated antibodies, anti-mouse (Invitrogen, 31438) or anti-rabbit (Invitrogen, 31458) were used, and either Enhanced ChemiLuminescence (Amersham, RPN2209) or Super Signal West Femto (Thermo Scientific, 34095) was used for detection. Dilution of all primary antibodies was 1:1,000 except GAPDH, vinculin and β-actin (1:10,000). Dilution of secondary antibodies was 1:10,000.

### Statistics and reproducibility

In general, at least two independent experiments were performed with similar results. Experiments included a minimum of three replicates as specified in figure legends. No statistical methods were used to predetermine sample size. Data distribution was assumed to be normal but this was not formally tested. No data were excluded from the analyses; the experiments were not randomized and the investigators were not blinded to allocation during experiments and outcome assessment. Comparisons between two groups were analyzed by two-tailed *t*-test, unequal variance or the MWW test. Results in graphs are expressed as mean ± s.d. or mean ± s.e.m. as indicated in figure legends. Box plots span the first to third quartiles and whiskers show 1.5× interquartile range. All statistical analyses were performed using GraphPad Prism software v.8.0 to obtain *P* values.

### Reporting summary

Further information on research design is available in the Nature Portfolio Reporting Summary linked to this article.

## Supplementary information

Reporting Summary

Supplementary Table 1 Supplementary Tables 1–12

## Data Availability

RNA-seq expression data of the 178 FFPE-derived and 45 frozen GBM IDH wild-type are available at Synapse (http://synapse.org; accession no. syn27042663). Previously published multi-omics data from CPTAC that were re-analyzed here are available from elswhere^6,46–48^. The human GBM transcriptomic, genomic, methylation and clinical data, BRCA and LUSC transcriptomic and clinical data were derived from the TCGA Research Network (http://cancergenome.nih.gov/) using TCGAbiolinks. BRCA transcriptomic data from METABRIC has been derived from elsewhere^63^. MNP-GBM methylation data were derived from the Gene Expression Omnibus (accession no. GSE90496). Source data have been provided as Source Data files. All other data supporting the findings of this study are available from the corresponding author on reasonable request.

## Source data

Source Data Fig. 4 Unprocessed western blots.
Source Data Fig. 6 Unprocessed western blots.
Source Data Fig. 6 Statistical Source Data.
Source Data Extended Data Fig. 6 Unprocessed western blots.
Source Data Extended Data Fig. 6 Statistical Source Data.

## Code availability

The source code used for SPHINKS and the GBM-specific kinome phosphorylome network are available at GitHub at https://github.com/miccec/MAKINA. The Shiny app of the frozen and FFPE classification tools is available at https://lucgar88.shinyapps.io/GBMclassifier.
