# Supplementary material for: Integrative multi-omics networks identify PKCδ and DNA-PK as master kinases of glioblastoma subtypes and guide targeted cancer therapy
Source: Nat Cancer. 2023 Feb 2;4(2):181–202. doi: 10.1038/s43018-022-00510-x (PMC9970878; doi:10.1038/s43018-022-00510-x)
Supplement: Supplementary file 1 — Reporting Summary [file 43018_2022_510_MOESM1_ESM.pdf]

nature portfolio

Corresponding author(s): Antonio Iavarone

Last updated by author(s): Dec 2, 2022

Reporting Summary

Nature Portfolio wishes to improve the reproducibility of the work that we publish. This form provides structure for consistency and transparency in reporting. For further information on Nature Portfolio policies, see our Editorial Policies and the Editorial Policy Checklist.

Statistics

For all statistical analyses, confirm that the following items are present in the figure legend, table legend, main text, or Methods section.

n/a	Confirmed	
☐	☒	The exact sample size (*n*) for each experimental group/condition, given as a discrete number and unit of measurement
☐	☒	A statement on whether measurements were taken from distinct samples or whether the same sample was measured repeatedly
☐	☒	The statistical test(s) used AND whether they are one- or two-sided<br>*Only common tests should be described solely by name; describe more complex techniques in the Methods section.*
☐	☒	A description of all covariates tested
☐	☒	A description of any assumptions or corrections, such as tests of normality and adjustment for multiple comparisons
☐	☒	A full description of the statistical parameters including central tendency (e.g. means) or other basic estimates (e.g. regression coefficient) AND variation (e.g. standard deviation) or associated estimates of uncertainty (e.g. confidence intervals)
☐	☒	For null hypothesis testing, the test statistic (e.g. *F*, *t*, *r*) with confidence intervals, effect sizes, degrees of freedom and *P* value noted<br>*Give P values as exact values whenever suitable.*
☒	☐	For Bayesian analysis, information on the choice of priors and Markov chain Monte Carlo settings
☐	☒	For hierarchical and complex designs, identification of the appropriate level for tests and full reporting of outcomes
☐	☒	Estimates of effect sizes (e.g. Cohen's *d*, Pearson's *r*), indicating how they were calculated

Our web collection on statistics for biologists contains articles on many of the points above.

Software and code

Policy information about availability of computer code

Data collection	No software was used
Data analysis	Blacksheepr R package v.1.2.0<br>glmnet R package v.4.1-2<br>gprofiler2 R package v.0.2.1<br>GraphPad Prism v.8.0.<br>ImageJ (Fiji) v.2.3.1<br>Ingenuity Pathways Knowledge Base QIAGEN IPA 2020 Release.<br>KinMap beta<br>MWW-GST v.2017.08.25.<br>R v.4.0.2<br>Set cover pathway redundancy v.1.<br>SNFtool R package v.2.3.1<br>TCGAbiolinks R package v.2.14.0.<br>DreamAI v.0.1.0<br><br>The source code used for SPHINKS and the GBM-specific kinome phosphorylome network are available at GitHub: https://github.com/miccec/MAKINA.<br>The Shiny app of the frozen and FFPE classification tools is available at https://lucgar88.shinyapps.io/GBMclassifier.

For manuscripts utilizing custom algorithms or software that are central to the research but not yet described in published literature, software must be made available to editors and reviewers. We strongly encourage code deposition in a community repository (e.g. GitHub). See the Nature Portfolio guidelines for submitting code & software for further information.

Data

Policy information about availability of data

All manuscripts must include a data availability statement. This statement should provide the following information, where applicable:

- Accession codes, unique identifiers, or web links for publicly available datasets
- A description of any restrictions on data availability
- For clinical datasets or third party data, please ensure that the statement adheres to our policy

RNA-Seq expression data of the 178 FFPE-derived and 45 frozen GBM IDH-wt tumors have been submitted to Synapse (http://synapse.org, accession no. syn27042663). Previously published multi-omics data from CPTAC that were re-analysed here are available from Ref 6, 46-48. The human GBM transcriptomic, genomic, methylation and clinical data, BRCA and LUSC transcriptomic and clinical data were derived from the TCGA Research Network: http://cancergenome.nih.gov/ using TCGAbiolinks. BRCA transcriptomic data from METABRIC has been derived from Ref 63. MolecularNeuroPathology (MNP) GBM methylation data were derived from GEO (accession no. GSE90496). Source data have been provided as Source Data files. All other data supporting the findings of this study are available from the corresponding author on reasonable request.
The list of figures that have associated biological raw data are:
Figure 4 d
Figure 6 a, b, c, d, e, f, g, h, i, j, k, l, m;
Extended Data Figure 6 b, c, d, e.

Human research participants

Policy information about studies involving human research participants and Sex and Gender in Research.

Reporting on sex and gender	Gender information is available in Supplementary Table 1 and 12. Analysis of breast carcinoma apply to female patients. Gender-based analysis were performed and reported in Figure 2.
Population characteristics	Studies include patient diagnosis with glioblastoma multiforme (adult), glioma (pediatric), breast carcinoma, lung adenocarcinoma. Age and diagnosis is reported in sSupplementary Table 1.<br><br>178 FFPE-derived and 45 frozen GBM IDH-wt tumors are from Onconeurotek tumor bank certification (NF S96 900) and authorization from Ethics committee (CPP Ile de France VI, ref A39II), and the French Ministry for research (AC 2013-1962) and were de-identified before reaching the research lab. Age and gender is reported in Supplementary Table 12.
Recruitment	Study does not involve recruitment procedures; this is exempt research.
Ethics oversight	Work with these materials was designated as IRB exempt under paragraph 4 and it is covered under IRB protocol and Onconeurotek tumor bank certification (NF S96 900) and authorization from Ethics committee (CPP Ile de France VI, ref A39II), and the French Ministry for research (AC 2013-1962).

Note that full information on the approval of the study protocol must also be provided in the manuscript.

Field-specific reporting

Please select the one below that is the best fit for your research. If you are not sure, read the appropriate sections before making your selection.

☒ Life sciences ☐ Behavioural & social sciences ☐ Ecological, evolutionary & environmental sciences

For a reference copy of the document with all sections, see nature.com/documents/nr-reporting-summary-flat.pdf

Life sciences study design

All studies must disclose on these points even when the disclosure is negative.

Sample size	Sample sizes were chosen based on data availability and on previous studies that showed robust statistical power. All available samples passing the quality control were included.
Data exclusions	No data were excluded.
Replication	At least three technical replicates were performed and experiments were repeated at least two times with similar results. All attempts at replication were successful.
Randomization	Our work does not include clinical or biospecimen-based studies and therefore there was no requirement for randomization in any of the experiments performed. For in-vitro experiments, randomization of cell lines was not possible. all cell lines were treated in same manner and when shRNA experiments were performed, all comparisons were between shRNA-PKCdelta and shRNA-NT to control the covariates.
Blinding	Molecular classification was performed independent of and blinded to the clinical features. Investigators were blinded to the clinical and

Blinding	molecular features during experiments and outcome assessments. For all in-vitro experiments, blinding is impossible as the same researcher need to treat the cells and run the analysis. However, quantification was automatically measured by plate reader. For IF pictures were coded prior to the data analysis.

Reporting for specific materials, systems and methods

We require information from authors about some types of materials, experimental systems and methods used in many studies. Here, indicate whether each material, system or method listed is relevant to your study. If you are not sure if a list item applies to your research, read the appropriate section before selecting a response.

Materials & experimental systems

n/a	Involved in the study	
☐	☒	Antibodies
☐	☒	Eukaryotic cell lines
☒	☐	Palaeontology and archaeology
☒	☐	Animals and other organisms
☒	☐	Clinical data
☒	☐	Dual use research of concern

Methods

n/a	Involved in the study	
☒	☐	ChIP-seq
☒	☐	Flow cytometry
☒	☐	MRI-based neuroimaging

Antibodies

Antibodies used

Anti- Akt (pan) (Cell Signaling Technology, #4691, C67E7, rabbit monoclonal, 1:1,000)
Anti- phospho-Akt (Cell Signaling Technology, #4060, Ser-473, D9E, rabbit monoclonal, 1:1,000)
Anti- phospho-Akt (Cell Signaling Technology, #13038, Thr-308, D25E6, rabbit monoclonal 1:1,000)
Anti- Stat3 (Cell Signaling Technology, #4904, 79D7, rabbit monoclonal 1:1,000)
Anti- phospho-Stat3 (Cell Signaling Technology, #9145, Tyr-705, D3A7, rabbit monoclonal, 1:1,000)
Anti- phospho-PKC Delta (Cell Signaling Technology, #2055, Tyr-311, rabbit polyclonal, 1:1,000)
Anti- PKC Delta (Abcam, #ab182126, EPR17075, rabbit monoclonal, 1:1,000)
Anti- PKC Delta (Cell Signaling Technology, #9616, D10E2, rabbit monoclonal, 1:1,000)
Anti- p44/42 MAPK (Erk1/2) (Cell Signaling Technology, #9102, rabbit polyclonal, 1:1,000)
Anti- phospho-p44/42 MAPK (Erk1/2) (Cell Signaling Technology, #4370, Thr202/Tyr204, D13.14.4E, rabbit monoclonal, 1:1,000)
Anti- phospho-DNA-PKcs (Cell Signaling Technology, #68716, Ser-2056, E9J4G, rabbit monoclonal, 1:1,000)
Anti- DNA-PKcs (Cell Signaling Technology, #38168, E6U3A, rabbit monoclonal, 1:1,000)
Anti- phospho-p95/NBS1 (Cell Signaling Technology, #3001, Ser-343, rabbit polyclonal, 1:1,000)
Anti- p95/NBS1 (Cell Signaling Technology, #14956, D6J5I, rabbit monoclonal, 1:1,000)
Anti- phospho-Histone H2A.X (Cell Signaling Technology, #2577, Ser-139, rabbit polyclonal, 1:1,000)
Anti- phospho-KAP1 (Abcam, #ab133440, Ser-824, EPR5248, rabbit monoclonal, 1:1,000)
Anti- KAP1 (Abcam, #ab109287, EPR5216, rabbit monoclonal, 1:1,000)
Anti- Chk1 (Cell Signaling Technology, #2360, 2G1D5, mouse monoclonal, 1:1,000)
Anti- phospho-Chk1 (Cell Signaling Technology, #12302, Ser-317, D12H3, rabbit monoclonal, 1:1,000)
Anti- β-actin (Sigma-Aldrich, #A5441, clone AC-15, mouse monoclonal, 1:10,000)
Anti- vinculin (Sigma-Aldrich, #V9131, clone hVIN-1, mouse monoclonal, 1:10,000)
Anti- GAPDH (Abcam, #ab9484, mAbcam 9484, mouse monoclonal, 1:10,000)
Anti-Mouse IgG (H+L) Cross-Adsorbed Secondary Antibody, HRP (Invitrogen, #31438, 1:10,000)
Anti-Rabbit IgG (H+L) Cross-Adsorbed Secondary Antibody, HRP (Invitrogen, #31458, 1:10,000)
Anti-Rabbit IgG (H+L) Cross-Adsorbed Secondary Antibody, Cyanine3 (Invitrogen, #A10520, 1:500)

Validation

Anti-Akt (pan) (Cell Signaling Technology, #4691, C67E7)
Reactivity: H/M/R/Mk/Dm, Sensitivity: endogenous, MW (kDa): 60, Source: rabbit monoclonal, Application-dilution: Western Blot-1:1,000/Immunoprecipitation- 1:50/Immunohistochemistry-1:150-1:600/Immunofluorescence-1:200-1:800/Flow Cytometry-1:100-1:400, Citation (PMID): 35415308, Akt (pan) (C67E7) Rabbit mAb detects endogenous levels of total Akt protein. This antibody does not cross-react with other related proteins. Monoclonal antibody is produced by immunizing animals with a synthetic peptide corresponding to residues in the carboxy-terminal sequence of mouse Akt. (https://www.cellsignal.com/products/primary-antibodies/akt-pan-c67e7-rabbit-mab/4691)
Anti-phospho-Akt (Cell Signaling Technology, #4060, Ser-473, D9E)
Reactivity: H/M/R/Hm/Mk/Dm/Z/B, Sensitivity: endogenous, MW (kDa): 60, Source: rabbit monoclonal, Application-dilution: Western Blot-1:2,000/Immunoprecipitation- 1:50/Immunohistochemistry-1:50-1:200/Immunofluorescence-1:400-1:800/Flow Cytometry-1:100-1:400, Citation (PMID): 35855640, Akt (pan) (C67E7) Rabbit mAb detects endogenous levels of total Akt protein. This antibody does not cross-react with other related proteins. Monoclonal antibody is produced by immunizing animals with a synthetic peptide corresponding to residues in the carboxy-terminal sequence of mouse Akt. (https://www.cellsignal.com/products/primary-antibodies/akt-pan-c67e7-rabbit-mab/4691)
Anti-Phospho-Akt (Cell Signaling Technology, #13038, Thr-308, D25E6)
Reactivity: H/M/R/Mk, Sensitivity: endogenous, MW (kDa): 60, Source: rabbit, Application-dilution: Western Blot-1:1,000/Immunoprecipitation- 1:50/Immunofluorescence-1:800 – 1:1600/Flow Cytometry-1:1600 – 1:6400, Citation (PMID): 36207295, Phospho-Akt (Thr308) (D25E6) XP® Rabbit mAb recognizes endogenous levels of Akt1 protein only when phosphorylated at Thr308. This antibody also recognizes endogenous levels of Akt2 protein when phosphorylated at Thr309 or Akt3 protein when phosphorylated at Thr305. Monoclonal antibody is produced by immunizing animals with a synthetic peptide corresponding to residues surrounding Thr308 of human Akt1 protein. (https://www.cellsignal.com/products/primary-antibodies/phospho-akt-thr308-d25e6-xp-rabbit-mab/13038).

Anti-Stat3 (Cell Signaling Technology, #4904, 79D7)
Reactivity: H/M/R/Mk, Sensitivity: endogenous, MW (kDa): 79/86, Source: rabbit monoclonal, Application-dilution: Western Blot-1:1,000/Immunoprcipitation-1:100/Chomatin IP: 1:50, Citation (PMID): 36289850, Stat3 (79D7) Rabbit mAb detects endogenous levels of total Stat3 protein. Monoclonal antibody is produced by immunizing animals with a Stat3 fusion protein corresponding to the carboxy-terminal sequence of mouse Stat3 protein. (https://www.cellsignal.com/products/primary-antibodies/stat3-79d7-rabbit-mab/4904).
Anti-Phospho-Stat3 (Cell Signaling Technology, #9145, Tyr-705, D3A7)
Reactivity: H/M/R/Mk, Sensitivity: endogenous, MW (kDa): 78/86, Source: rabbit, Application-dilution: Western Blot-1:1,000/ Immunoprecipitation- 1:100/IHC-Leica BOND- 1:100 – 1:400/Immuohistochemistry- 1:100 – 1:400/Immunofluorescence-1:100 – 1:200/ Flow Cytometry-1:100 – 1:400/Chromatin IP- 1:100/Chromatin IP-seq- 1:100, Citation (PMID): 35896788, Phospho-Stat3 (Tyr705) (D3A7) XP® Rabbit mAb detects endogenous levels of Stat3 only when phosphorylated at tyrosine 705. This antibody does not cross-react with phospho-EGFR or the corresponding phospho-tyrosines of other Stat proteins. Monoclonal antibody is produced by immunizing animals with a synthetic phosphopeptide corresponding to residues surrounding Tyr705 of mouse Stat3. (https://www.cellsignal.com/products/primary-antibodies/phospho-stat3-tyr705-d3a7-xp-rabbit-mab/9145).
Anti-phospho-PKCδ (Cell Signaling Technology, #2055, Tyr-311)
Reactivity: H/M/R, Sensitivity: endogenous, MW (kDa): 80, Source: rabbit, Application-dilution: Western Blot-1:1,000, Citation (PMID): 35166238, Phospho-PKCdelta (Tyr311) Antibody detects endogenous levels of PKCdelta only when phosphorylated at tyrosine 311. This antibody does not cross-react with other phosphorylated PKC isoforms. Polyclonal antibodies are produced by immunizing animals with a synthetic phosphopeptide corresponding to residues surrounding Tyr313 of human PKCdelta (which is equivalent to Tyr311 in mouse and rat). Antibodies are purified by protein A and peptide affinity chromatography. (https://www.cellsignal.com/products/primary-antibodies/phospho-pkcdelta-tyr311-antibody/2055).
Anti-PKC Delta (Abcam, #ab182126, EPR17075)
Reactivity: Mouse/Rat/Human, MW (kDa): 78, Source: rabbit monoclonal, Application-dilution: Flow Cyt-1:250/IHC-P-1:2,000/ WB-1:5,000/ICC/IF-5 µg/ml, Citation (PMID): 33688230, Recombinant fragment. This information is proprietary to Abcam and/or its suppliers. (https://www.abcam.com/pkc-delta-antibody-epr17075-ab182126.html).
Anti-PKC Delta (Cell Signaling Technology, #9616, D10E2)
Reactivity: H/M/R/Mk Sensitivity: endogenous, MW (kDa): 78, Source: rabbit monoclonal, Application-dilution: Western Blot-1:1,000/ Immunoprecipitation-1:50, Citation (PMID): 30979895, PKCδ (D10E2) Rabbit mAb recognizes endogenous levels of total PKCδ protein. This antibody does not cross-react with other PKC isoforms. Monoclonal antibody is produced by immunizing animals with a synthetic peptide corresponding to residues surrounding Arg216 of human PKCδ protein. (https://www.cellsignal.com/products/primary-antibodies/pkcd-d10e2-rabbit-mab/9616).
Anti-p44/42 MAPK (Erk1/2) (Cell Signaling Technology, #9102)
Reactivity: H/M/R/Mk/Mi/Z/B/Pg/Sc Sensitivity: endogenous, MW (kDa): 42/44, Source: rabbit, Application-dilution: Western Blot-1:1,000/Immunoprecipitation-1:50/Immunohistochemistry- 1:50 – 1:200, Citation (PMID): 36336784, p44/42 MAPK (Erk1/2) Antibody detects endogenous levels of total p44/42 MAP kinase (Erk1/Erk2) protein. In some cell types, this antibody recognizes p44 MAPK more readily than p42 MAPK. The antibody does not recognize either JNK/SAPK or p38 MAP kinase. Polyclonal antibodies are produced by immunizing animals with a synthetic peptide corresponding to a sequence in the C-terminus of rat p44 MAP Kinase. Antibodies are purified by protein A and peptide affinity chromatography. (https://www.cellsignal.com/products/primary-antibodies/p44-42-mapk-erk1-2-antibody/9102).
Anti-phospho-p44/42 MAPK (Erk1/2) (Cell Signaling Technology, #4370, Thr202/Tyr204, D13.14.4E)
Reactivity: H/M/R/Mk/Mi/Dm/Z/B/Dg/Pg/Sc, Sensitivity: endogenous, MW (kDa): 42/44, Source: rabbit monoclonal, Application-dilution: Western Blot-1:2,000/Immunoprecipitation- 1:50/Immunohistochemistry - 1:200 – 1:400/Immunofluorescence-1:200 – 1:400/Flow Cytometry-1:800 – 1:1600, Citation (PMID): 36376983, Phospho-p44/42 MAPK (Erk1/2) (Thr202/Tyr204) (D13.14.4E) XP® Rabbit mAb detects endogenous levels of p44 and p42 MAP Kinase (Erk1 and Erk2) when dually phosphorylated at Thr202 and Tyr204 of Erk1 (Thr185 and Tyr187 of Erk2), and singly phosphorylated at Thr202. This antibody does not cross-react with the corresponding phosphorylated residues of either JNK/SAPK or p38 MAP kinases. Monoclonal antibody is produced by immunizing animals with a synthetic phosphopeptide corresponding to residues surrounding Thr202/Tyr204 of human p44 MAP kinase. (https://www.cellsignal.com/products/primary-antibodies/phospho-p44-42-mapk-erk1-2-thr202-tyr204-d13-14-4e-xp-rabbit-mab/4370).
Anti-phospho-DNA-PKcs (Cell Signaling Technology, #68716, Ser2056, E9J4G)
Reactivity: H, Sensitivity: endogenous, MW (kDa): 450, Source: rabbit monoclonal, Application-dilution: Western Blot-1:1,000, Citation (PMID): 34644577, Phospho-DNA-PKcs (Ser2056) (E9J4G) Rabbit mAb recognizes endogenous levels of DNA-PKcs protein only when phosphorylated at Ser2056. Monoclonal antibody is produced by immunizing animals with a synthetic peptide corresponding to residues surrounding Ser2056 of human DNA-PKcs protein. (https://www.cellsignal.com/products/primary-antibodies/phospho-dna-pkcs-ser2056-e9j4g-rabbit-mab/68716)
Anti-DNA-PKcs (Cell Signaling Technology, #38168, E6U3A)
Reactivity: H, Sensitivity: endogenous, MW (kDa): 450, Source: rabbit monoclonal, Application-dilution: Western Blot-1:1,000/ Immunohistochemistry-1:800/Immunofluorescence-1:100/Flow Cytometry-1:100, Citation (PMID): 35173610, DNA-PKcs (E6U3A) Rabbit mAb recognizes endogenous levels of total DNA-PKcs protein. Monoclonal antibody is produced by immunizing animals with a synthetic peptide corresponding to residues surrounding Pro608 of human DNA-PKcs protein. (https://www.cellsignal.com/products/primary-antibodies/dna-pkcs-e6u3a-rabbit-mab/38168)
Anti-phospho-p95/NBS1 (Cell Signaling Technology, #3001, Ser-343)
Reactivity: H, Sensitivity: endogenous, MW (kDa): 95, Source: rabbit, Application-dilution: Western Blot-1:1,000, Citation (PMID): 36242003, Phospho-p95/NBS1 (Ser343) Antibody detects endogenous levels of p95/NBS1 only when phosphorylated at serine 343. Polyclonal antibodies are produced by immunizing animals with a synthetic phosphopeptide corresponding to residues surrounding Ser343 of human p95/NBS1. Antibodies are purified by protein A and peptide affinity chromatography. (https://www.cellsignal.com/products/primary-antibodies/phospho-p95-nbs1-ser343-antibody/3001)
Anti-p95/NBS1 (Cell Signaling Technology, #14956, D6J5I)
Reactivity: H/M/R, Sensitivity: endogenous, MW (kDa): 95, Source: rabbit monoclonal, Application-dilution: Western Blot-1:1,000/ Immunoprcipitation-1:100/Immunofluorescence-1:100, Citation (PMID): 35551189, p95/NBS1 (D6J5I) Rabbit mAb recognizes endogenous levels of total p95/NBS1 protein. This antibody also cross-reacts with an unidentified protein of 180 kDa in some cell lines. Monoclonal antibody is produced by immunizing animals with a synthetic peptide corresponding to residues surrounding Ala740 of human p95/NBS1 protein. (https://www.cellsignal.com/products/primary-antibodies/p95-nbs1-d6j5i-rabbit-mab/14956)
Anti-phospho-Histone H2A.X (Cell Signaling Technology, #2577, Ser-139)
Reactivity: H/M/R/Mk, Sensitivity: endogenous, MW (kDa): 15, Source: rabbit, Application-dilution: Western Blot-1:1,000/ Immunofluorescence-1:400 – 1:1600/Flow Cytometry-1:200, Citation (PMID): 36092604, Phospho-H2A.X (Ser139) Antibody detects endogenous levels of H2A.X only when phosphorylated at Ser139. Antibodies are produced by immunizing animals with a synthetic

phosphopeptide corresponding to residues surrounding Ser139 of human H2A.X. (https://www.cellsignal.com/products/primary-antibodies/phospho-histone-h2a-x-ser139-antibody/2577)
Anti-phospho-KAP1 (Abcam, #ab133440, Ser-824, EPR5248)
Reactivity: Mouse/Human, MW (kDa): 88, Source: rabbit monoclonal, Application-dilution: WB-1:1,000/IP-1:10-1:100, Citation (PMID): 34108527, Synthetic peptide. This information is proprietary to Abcam and/or its suppliers. (https://www.abcam.com/kap1-phospho-s824-antibody-epr5248-ab133440.html).
Anti-KAP1 (Abcam, #ab109287, EPR5216)
Reactivity: Mouse/Human, MW (kDa): 89, Source: rabbit monoclonal, Application-dilution: WB-1:10,000-1:50,000/IHC-P-1:250-1:500/ICC/IF-1:100-1:250, Citation (PMID): 36198274, Synthetic peptide. This information is proprietary to Abcam and/or its suppliers. (https://www.abcam.com/kap1-antibody-epr5216-ab109287.html).
Anti-Chk1 (Cell Signaling Technology, #2360, 2G1D5)
Reactivity: H/M/R/Mk Sensitivity: endogenous, MW (kDa): 56, Source: mouse monoclonal, Application-dilution: Western Blot-1:1,000, Citation (PMID): 36266721, Chk1 (2G1D5) Mouse mAb recognizes endogenous levels of total Chk1 protein. Monoclonal antibody is produced by immunizing animals with purified recombinant Chk1 protein. (https://www.cellsignal.com/products/primary-antibodies/chk1-2g1d5-mouse-mab/2360)
Anti-phospho-Chk1(Cell Signaling Technology, #12302, Ser-317, D12H3)
Reactivity: H/M/Mk Sensitivity: endogenous, MW (kDa): 56, Source: rabbit monoclonal, Application-dilution: Western Blot-1:1,000/Immunoprcipitation-1:50/Immunofluorescence-1:800- 1:1600, Citation (PMID): 36123339, Phospho-Chk1 (Ser317) (D12H3) XP® Rabbit mAb recognizes endogenous levels of Chk1 protein only when phosphorylated at Ser317. This antibody also detects an 80 kDa protein of unknown origin in some cell lines. Monoclonal antibody is produced by immunizing animals with a synthetic phosphopeptide corresponding to residues surrounding Ser317 of human Chk1 protein. (https://www.cellsignal.com/products/primary-antibodies/phospho-chk1-ser317-d12h3-xp-rabbit-mab/12302)
Anti-β-actin (Sigma-Aldrich, #A5441, clone AC-15)
Reactivity: human/bovine/sheep/pig/rabbit/cat/dog/mouse/rat/guinea pig/chicken/carp/leech tissues, MW (kDa): 42, Source: mouse, Application-dilution: Immunoblotting-1:5,000/Indirect immunofluorescence-1:1,000, Citation (PMID): 8436588, Monoclonal Anti-b-Actin (mouse IgG1 isotype) is derived from the AC-15 hybridoma produced by the fusion of mouse myeloma cells and splenocytes from an immunized mouse. A slightly modified synthetic b-cytoplasmic actin N-terminal peptide Ac-Asp-Asp-Asp-Ile-Ala-Ala-Leu-Val-Ile-Asp-Asn-Gly-Ser-Gly-Lys conjugated to KLH was used as the immunogen. The isotype is determined by a double diffusion immunoassay using Mouse Monoclonal Antibody Isotyping Reagents, Catalog Number ISO2. (https://www.sigmaaldrich.com/US/en/product/sigma/a5441)
Anti-vinculin (Sigma-Aldrich, #V9131, clone hVIN-1)
Reactivity: human/bovine/chicken/dog/rat/mouse/turkey/xenopus,/smooth muscle metavinculin, MW (kDa): 116, Source: mouse, Application-dilution: A minimum antibody titer of 1:400 is determined by indirect immunofluorescent labeling of cultured human fibroblasts. In order to obtain best results in various techniques and preparations, it is recommended that each individual user determines their optimum working dilution by titration, Citation (PMID): 2116004, Monoclonal Anti-Vinculin (mouse IgG1 isotype) is derived from the hVIN-1 hybridoma produced by the fusion of mouse myeloma cells and splenocytes from immunized BALB/c mice. Vinculin, purified from human uterus, was used as the immunogen. The isotype is determined using Sigma ImmunoTypeTM Kit (Product Code ISO-1) and by a double diffusion immunoassay using Mouse Monoclonal Antibody Isotyping Reagents (Product Code ISO-2). (https://www.sigmaaldrich.com/US/en/product/sigma/v9131)
Anti-GAPDH (Abcam, #ab9484, mAbcam 9484)
Reactivity: Mouse/Rat/Chicken/Cow/Human/Pig/Xenopus laevis/Chinese hamster, MW (kDa): 36, Source: mouse, Application-dilution: WB- 0.1 - 1 µg/ml/IHC-P- 5 µg/ml, Citation (PMID): 33264494, Full length native protein (purified). This information is proprietary to Abcam and/or its suppliers. (https://www.abcam.com/gapdh-antibody-mabcam-9484-loading-control-ab9484.html).
Anti-Mouse IgG (H+L) Cross-Adsorbed Secondary Antibody, HRP (Invitrogen, #31438)
Reactivity: This antibody reacts with the heavy chains on mouse IgG and with the light chains common to most mouse immunoglobulins. No antibody was detected against non-immunoglobulin serum proteins. The antibody has been tested by ELISA and/or solid-phase adsorbed to ensure minimal cross-reaction with human, bovine and horse serum proteins. However, this antibody may cross-react with immunoglobulins from other species., Application-dilution: Western Blot-1:10,000-1:200,000/Immunohistochemistry-1:500-1:5,000/Immunocytochemistry-1:500-1:5,000, Citation (PMID): 35991835, This antibody has been isolated from antisera by combination of pepsin digestion and immunoaffinity chromatography, using antigen coupled to agarose beads. Fc fragments and whole IgG molecules have been removed. (https://www.thermofisher.com/antibody/product/Goat-anti-Mouse-IgG-H-L-Cross-Adsorbed-Secondary-Antibody-Polyclonal/31438)
Anti-Rabbit IgG (H+L) Cross-Adsorbed Secondary Antibody, HRP (Invitrogen, #31458)
Reactivity: This antibody reacts with the heavy chains of rabbit IgG and with the light chains common to most rabbit immunoglobulins. No antibody was detected against non-immunoglobulin serum proteins. The antibody has been tested by ELISA and/or solid-phase adsorbed to ensure minimal cross-reaction with bovine, chicken, goat, guinea pig, hamster, horse, human, mouse, rat and sheep serum proteins. However, this antibody may cross-react with immunoglobulins from other species., Application-dilution: Western Blot-1:10,000-1:200,000/Immunohistochemistry-1:500-1:5,000/Immunocytochemistry-1:500-1:5,000, Citation (PMID): 36244455. (https://www.thermofisher.com/antibody/product/Donkey-anti-Rabbit-IgG-H-L-Cross-Adsorbed-Secondary-Antibody-Polyclonal/31458)
Anti-Rabbit IgG (H+L) Cross-Adsorbed Secondary Antibody, Cyanine3 (Invitrogen, #A10520)
Application-dilution: Western Blot-1:10,000/Immunocytochemistry-1-10 µg/mL, Citation (PMID): 36424632, Immunogen: Gamma Immunoglobins Heavy and Light chains. (https://www.thermofisher.com/antibody/product/Goat-anti-Rabbit-IgG-H-L-Cross-AdsorbedSecondary-Antibody-Polyclonal/A10520)

The following are the Research Resource Identifiers (RRIDs) from the Resource Identification Portal, supporting guidelines for Rigor and Transparency in scientific publications.
Anti- Akt (pan), RRID:AB_915783
Anti- phospho-Akt, RRID:AB_331170
Anti- phospho-Akt, RRID:AB_2629447
Anti- Stat3, RRID:AB_331269
Anti- phospho-Stat3, RRID:AB_2491009
Anti- phospho-PKC Delta, RRID:AB_330876
Anti- PKC Delta, RRID:AB_2892154
Anti- PKC Delta, RRID:AB_10949973
Anti- p44/42 MAPK (Erk1/2), RRID:AB_330744

Anti- phospho-p44/42 MAPK (Erk1/2), RRID:AB_2315112
Anti- DNA-PKcs, RRID:AB_2799128
Anti- phospho-p95/NBS1, RRID:AB_10829154
Anti- p95/NBS1, RRID:AB_2798660
Anti- phospho-Histone H2A.X, RRID:AB_2118010
Anti- KAP1, RRID:AB_10858772
Anti- Chk1, RRID:AB_2080320
Anti- phospho-Chk1, RRID:AB_2783865
Anti- β-actin, RRID:AB_476744
Anti- Vinculin, RRID:AB_477629
Anti- GAPDH, RRID:AB_307274
Anti-Mouse IgG (H+L) Cross-Adsorbed Secondary Antibody, RRID:AB_228217
Anti-Rabbit IgG (H+L) Cross-Adsorbed Secondary Antibody, RRID:AB_228213
Anti-Rabbit IgG (H+L) Cross-Adsorbed Secondary Antibody, Cyanine3, RRID:AB_2534029
Anti-phospho-DNA-PKcs (https://www.cellsignal.com/products/primary-antibodies/phospho-dna-pkcs-ser2056-e9j4g-rabbit-mab/68716) and Anti-phospho-KAP1 (https://www.abcam.com/kap1-phospho-s824-antibody-epr5248-ab133440.html) were validated by published studies (Anti-phospho-DNA-PKcs, PMID: 34644577; Anti-phospho-KAP1: PMID: 34108527).

Eukaryotic cell lines

Policy information about cell lines and Sex and Gender in Research

Cell line source(s)

HEK293T (ATCC CRL-11268).

Patient-derived organoids (PDOs) were obtained using excess material collected for clinical purposes from de-identified brain tumor specimens. Donors (patients diagnosed with glioblastoma) were anonymous. Progressive numbers were used to label specimens coded in order to preserve the confidentiality of the subjects. Work with these materials was designated as IRB exempt under paragraph 4 and it is covered under IRB protocol #IRB-AAAI7305 and Onconeurotek tumor bank certification (NF S96 900) and authorization from Ethics committee (CPP Ile de France VI, ref A39II), and the French Ministry for research (AC 2013-1962). Of the 23 PDOs utilized in the study 14 were males and 9 were females as assessed by the analysis of chromosome X and Y ratio from whole exome sequencing.

Authentication

Cell authentication was performed using short tandem repeats (STR) at the ATCC facility.

Mycoplasma contamination

Cells were routinely tested for mycoplasma contamination using the Mycoplasma Plus PCR Primer Set (Agilent Technologies) and were found to be negative.

Commonly misidentified lines
(See ICLAC register)

We have not used cell lines listed in the database of commonly misidentified cell lines.
