# Supplementary figures and images for: Integrative multi-omics networks identify PKCδ and DNA-PK as master kinases of glioblastoma subtypes and guide targeted cancer therapy
Source: Nat Cancer. 2023 Feb 2;4(2):181–202. doi: 10.1038/s43018-022-00510-x (PMC9970878; doi:10.1038/s43018-022-00510-x)

**p-Ser-317-CHK1**

136 kD  
100 kD

**Vinculin**

136 kD  
100 kD

**CHK1**

75 kD  
63 kD

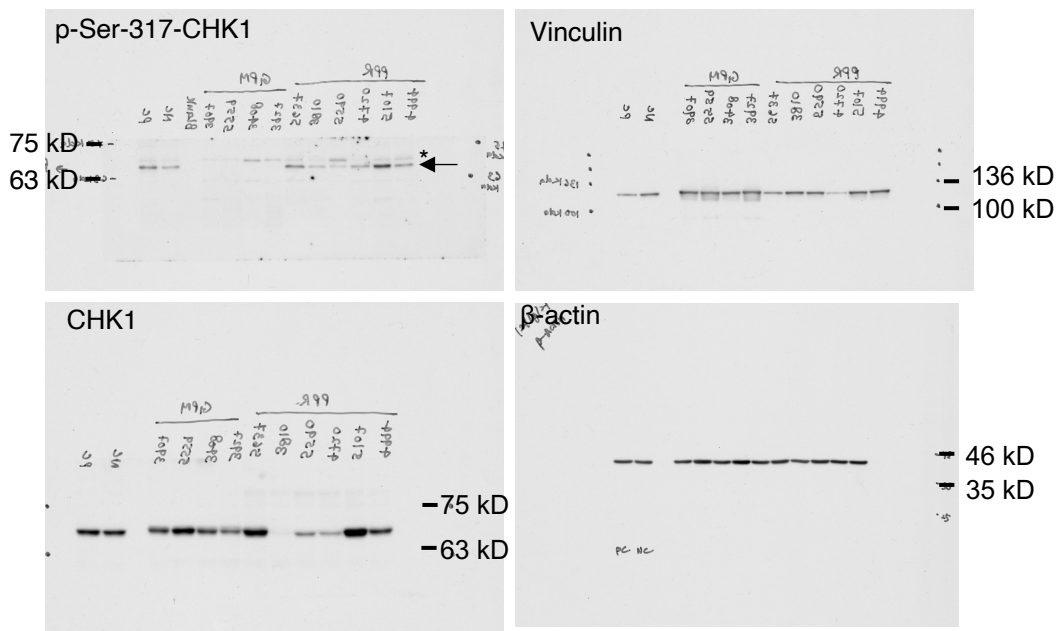

Supplement: Source Data Fig. 4 — Unprocessed western blots. [file 43018_2022_510_MOESM3_ESM.pdf]

Figure 6d

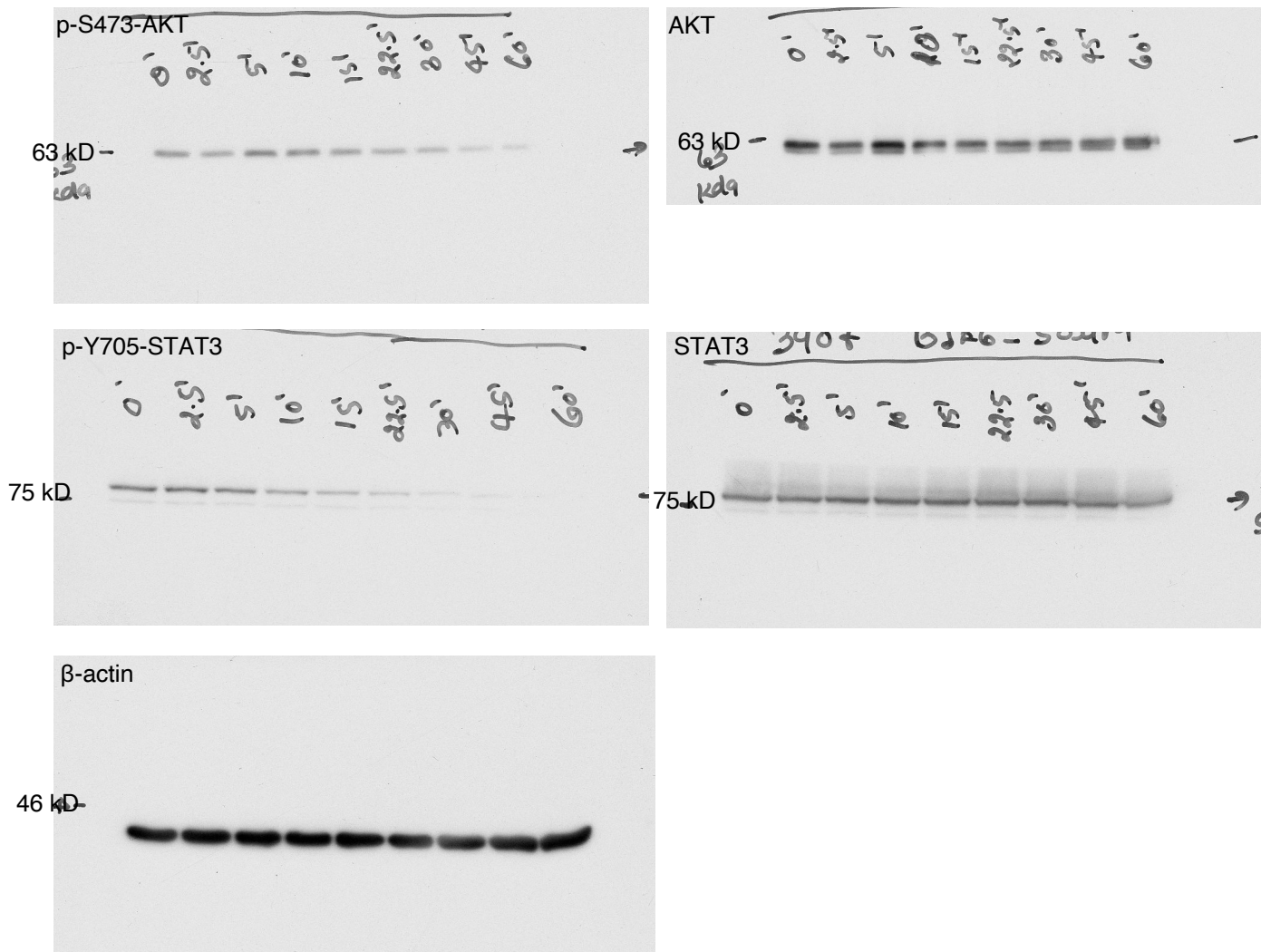

Figure 6e

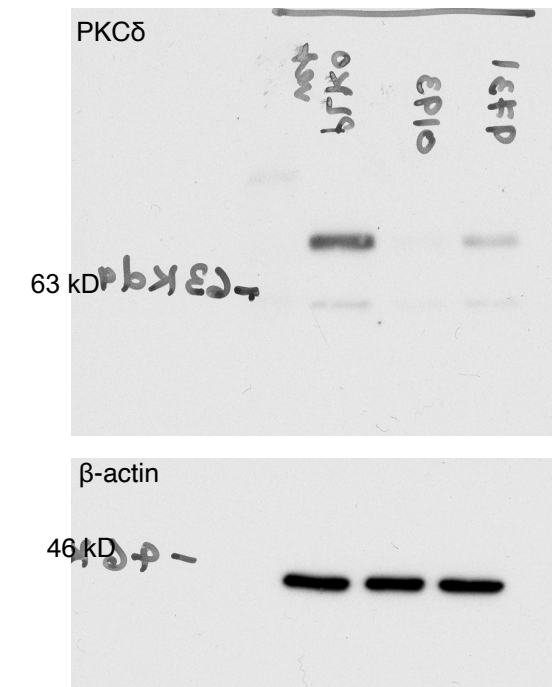

Figure 6I

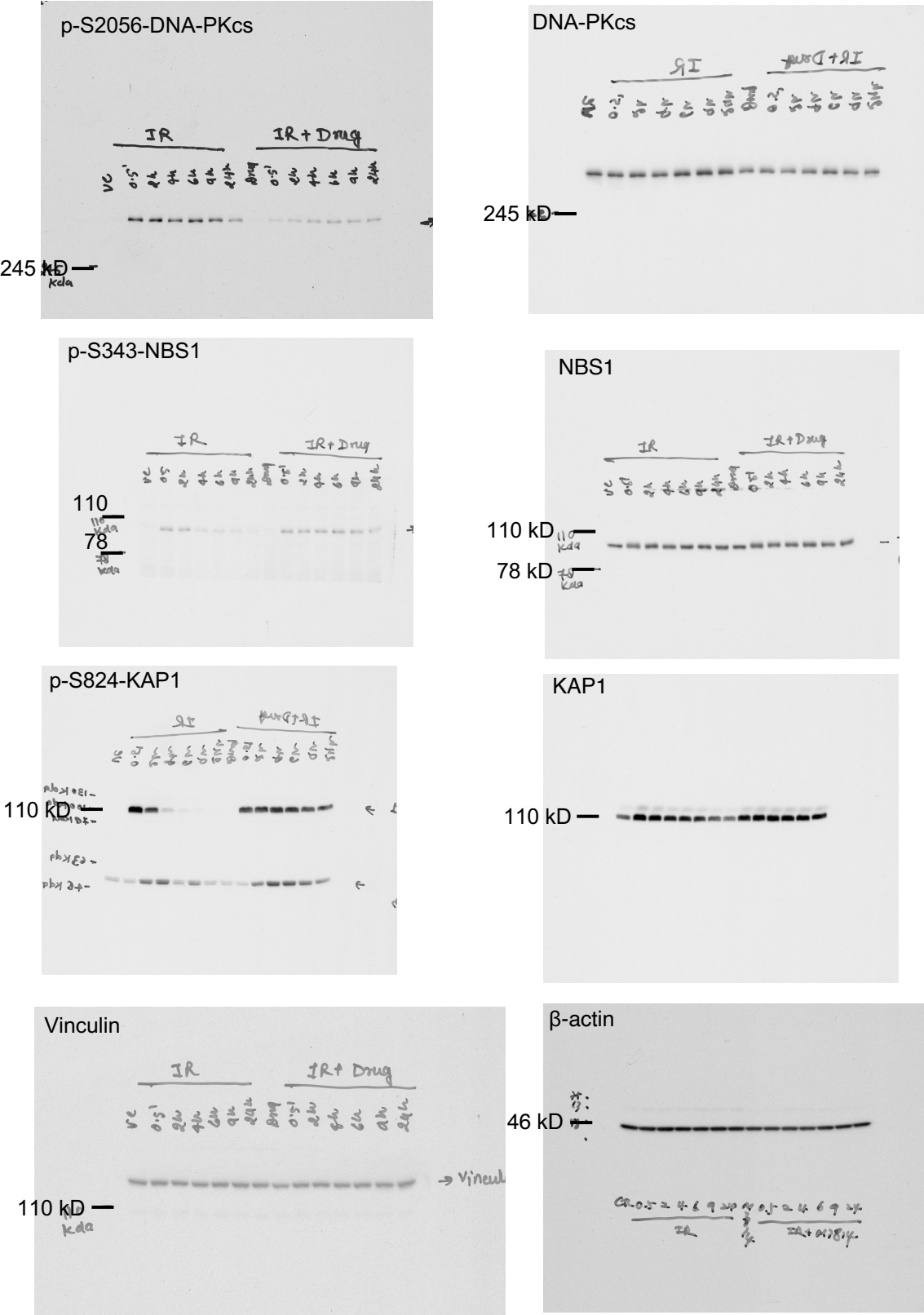

Supplement: Source Data Fig. 6 — Unprocessed western blots. [file 43018_2022_510_MOESM4_ESM.pdf]

ED Figure 6b

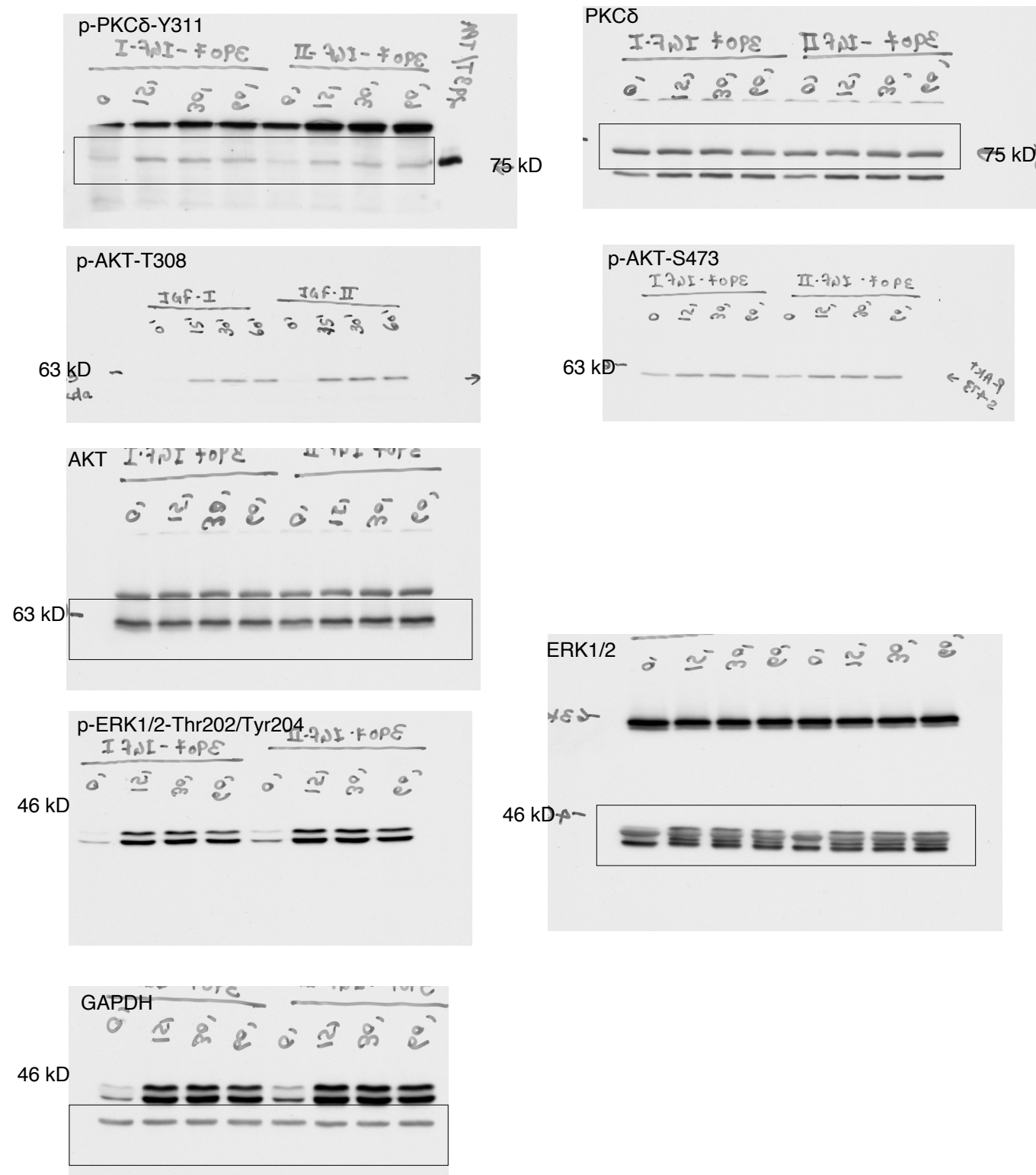

ED Figure 6c

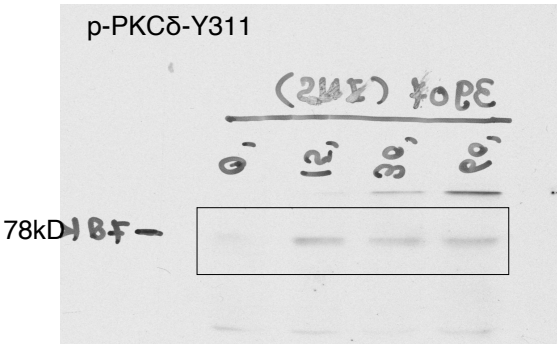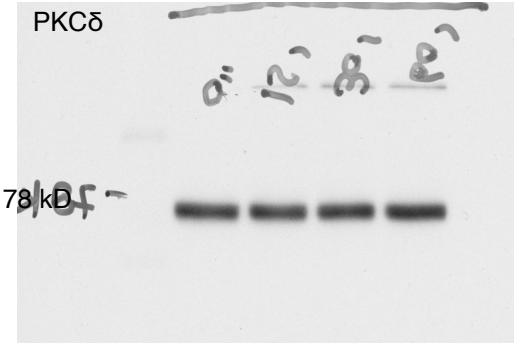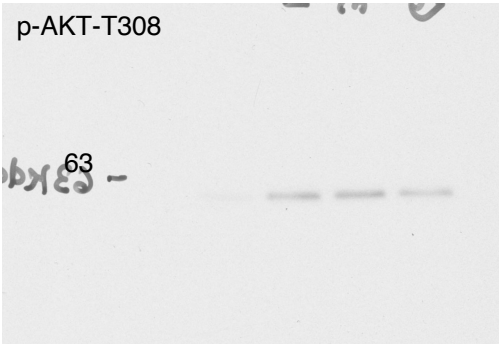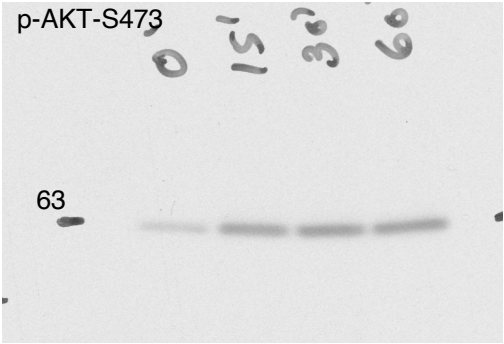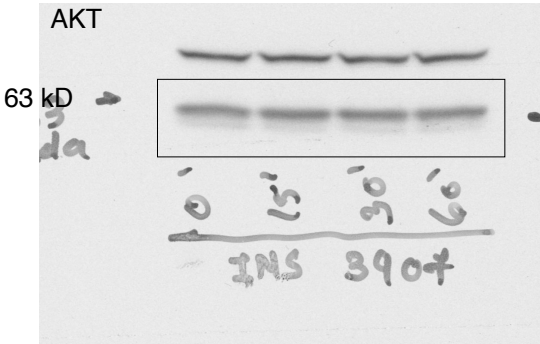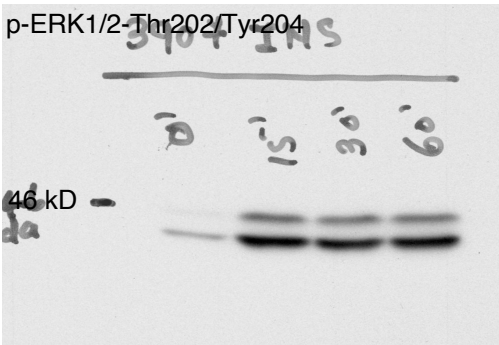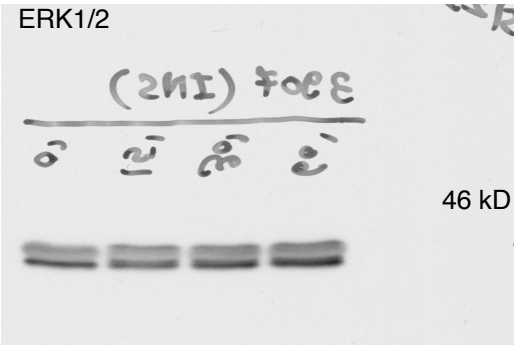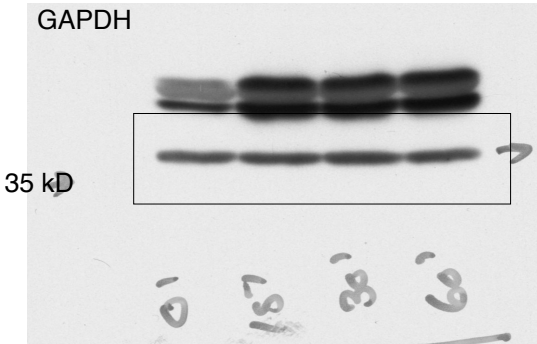

Supplement: Source Data Extended Data Fig. 6 — Unprocessed western blots. [file 43018_2022_510_MOESM6_ESM.pdf]
